# “A tool in a toolbox”: patient engagement with a gamified and personalised approach bias modification app to reduce harmful alcohol consumption – a qualitative study

**DOI:** 10.1186/s13722-026-00646-6

**Published:** 2026-01-31

**Authors:** Victoria Manning, Mietta C. L. Bell, Joshua B. B. Garfield, Josephine C. B. Paxie, Adam Rubenis, Ariel D. Roxburgh, Hugh Piercy, Danielle Whelan, Dan I. Lubman, Michael Savic

**Affiliations:** 1 Monash Addiction Research Centre, Eastern Health Clinical School, Monash University, Turning Point, 110 Church Street, Richmond, VIC 3121 Australia; 2 Turning Point, Eastern Health, 110 Church Street, Richmond, VIC 3121 Australia

**Keywords:** Approach bias modification, Cognitive bias modification, Alcohol use disorder, Smartphone app, Qualitative research, Addiction treatment, Gender

## Abstract

**Background:**

Approach Bias Modification (ApBM) is a recommended adjunctive intervention during residential treatment for Alcohol Use Disorder (AUD), and there are also promising findings for ApBM as an adjunctive intervention for people in outpatient treatment. ApBM can be delivered via smartphone apps, yet little is known about how people interact with and interpret ApBM apps. We adopted an ‘evidence-making intervention’ approach to qualitatively explore experiences of an AUD ApBM app, to guide implementation in real-world settings. Whilst evidence-based research is mostly concerned with whether trials show that ApBM reduces approach bias and relapse/alcohol use, evidence-making research is concerned with *how* things like trial design, measures, contexts, and narratives of participants shape our understanding of ApBM’s effectiveness.

**Methods:**

20 participants (12 men, 8 women; mean age 52.1 years) from two randomised controlled trials of a personalised and gamified ApBM app (10 outpatient, 10 residential rehabilitation) completed semi-structured phone interviews exploring their experiences with the app, its perceived effects, and its alignment with other treatment options. Common themes were identified using thematic analysis.

**Results:**

Participants engaged with the app in several ways, with four primary themes identified. The first, context and structure of app use (part of daily routines), with preferred times of day and locations for use, to support habit formation. Second, gendered responses to gamification elements shaped participant engagement with the intervention, with male participants framing it more as a game, and females tending to describe it as monotonous. The third theme encompassed participants’ multiple perceptions of how the app works, including as: a craving distraction tool; reinforcing recovery goals and life beyond alcohol use; assisting with countering the effects of environmental alcohol-related triggers; and affecting subconscious processes. The fourth theme related to participants’ feelings that the app was complementary to other addiction treatment and support.

**Conclusions:**

Participants interpreted and applied ApBM in ways that went beyond the primary intentions of the app, with most perceiving it as a useful supplement to other forms of AUD support. These findings highlight the importance of exploring user experiences and incorporating consumer feedback in future app design to improve the implementation of this brief, convenient, low-cost intervention.

**Trial registration:**

The Outpatient project was prospectively registered with ClinicalTrials.gov on November 11, 2021, registration number NCT05120856 https://clinicaltrials.gov/ct2/show/NCT05120856. The Rehabilitation project was prospectively registered with the Australian New Zealand Clinical Trials Registry on September 15 2022, registration number ACTRN12622001245785 https://anzctr.org.au/Trial/Registration/TrialReview.aspx?ACTRN=12622001245785.

**Supplementary Information:**

The online version contains supplementary material available at 10.1186/s13722-026-00646-6.

## Background

Approach Bias Modification (ApBM) is a form of computerised neurocognitive training that aims to retrain the subconscious and automatic action tendency to approach alcohol-related cues, which is thought to contribute to the maintenance of alcohol use disorder (AUD) [1]. ApBM requires users to repeatedly push away images of alcohol and approach non-alcohol/neutral images using a joystick and computer, such that the avoidance of alcohol becomes a more automatic response. Several double-blind randomised controlled trials (RCTs) show that adding ApBM during residential treatment significantly reduces the likelihood of relapse to drinking post-discharge relative to a control condition [2–7]. Based on this evidence, a global Delphi consensus study identified ApBM as an effective treatment for AUD [8]. ApBM is now recommended as an adjunctive intervention for people undergoing residential AUD treatment in both Australian and German alcohol treatment guidelines [9, 10].

Recognising the need to expand ApBM adjunctive treatment beyond residential settings, and to the broader population of non-treatment seekers with alcohol problems, researchers have begun developing smartphone-delivered ApBM interventions. A Dutch trial of the “Breindebaas” app [11] and an Australian trial of the “SWiPE” app [12] reported similar reductions in weekly use (~ 8 standard drinks) after 3 or 4 weeks of app use in large (n *≥* 1000) community samples comprised largely of non-treatment-seekers. Whilst these results are promising, neither study included a control condition, highlighting the need to rigorously examine the efficacy of ApBM apps in both non-treatment and treatment-seeking populations.

Our team recently completed two (RCTs) using an improved version of the personalised and gamified SWiPE app, called “AAT-App” (Alcohol Avoidance Training App). One trial recruited people who were accessing outpatient treatment for alcohol problems [13], while the second provided the app to patients after their discharge from inpatient residential rehabilitation [14]. Participants were trained to avoid (swipe away) personalised images of alcohol (i.e., beverages selected by the participant prior to training to represent drinks they typically consumed) and to approach (swipe towards) personally-selected images that represented their goals, hobbies, interests or values. Participants were encouraged to use the app for four weeks.

In the trial of AAT-App with outpatients [13], significant group differences were not observed in mean weekly standard drinks immediately post-intervention (4-week follow-up) relative to controls who received sham-training. However, at a 16-week follow-up, ApBM participants showed a significantly larger decrease than controls in past-week standard drinks, relative to their baseline levels (reduction of 14.6 standard drinks in ApBM group versus 2.1 in controls, *p* = 0.03). Results of the rehabilitation trial [14] are not yet published.

In both outpatient and inpatient trials, we conducted qualitative interviews with participants to understand how people experience, use, and understand ApBM. Qualitative research aims to deepen an understanding of the phenomenon being studied and the social world of participants, rather than simple measurement of outcomes [15]. There remains a dearth of qualitative research on cognitive bias modification treatments more broadly [16]. Such research is critical for understanding the personal and contextual factors that influence the adoption of, and adherence to, mobile health (m-health) interventions, and to inform further design and development that optimises engagement and effectiveness.

To date, there have only been two qualitative studies of ApBM interventions for alcohol. The first, conducted among treatment-seeking young adults with harmful alcohol use and symptoms of social anxiety (*N* = 15), found good acceptability of the computer program, which combined ApBM with interpretation bias modification. Themes encompassed ease of use, appropriateness, clinical value, engagement/motivation, and various suggestions for improvements [17]. The only qualitative study of an ApBM app for alcohol use involved non-treatment seekers (*N* = 18) who used the SWiPE app [18]. Findings illustrated marked heterogeneity in its perceived effectiveness, how it was understood to work, and the various ways participants incorporated it into their lives. For example, some participants described integrating the app into established daily routines as part of a broader ‘habit-stacking’ approach [19], using it alongside other health-promoting behaviours to build consistency and reinforce positive change. Other participants described using it more reactively as an ‘on-demand circuit-breaker’, engaging with the app in moments of acute craving to interrupt automatic drinking impulses and regain a sense of control by increasing the time between alcohol craving and seeking. However, it is unclear whether these findings, based on data from people drinking at hazardous levels in the general community who were not accessing formal treatment for AUD, would generalise to people accessing treatment for AUD. Treatment-seekers often have more severe AUD than general community samples, and would be using ApBM alongside other supports and interventions, rather than in isolation as a stand-alone tool. Exploring how ApBM is understood, used, and experienced by people in treatment for AUD, its perceived compatibility with concurrent treatment, and its safety, is key to guiding the future implementation of this ultra-brief, convenient, low-cost and accessible intervention in treatment contexts/populations. It is particularly valuable to understand the many ways apps materialise in treatment contexts, which are often complex; involve various individuals, as well as social and gendered dynamics; and extend beyond the control of app developers. This could reveal unexpected and diverse forms of both app engagement and app effects. Since ApBM purportedly aims to target subconscious, automatic processes, rich user insights into factors that promote or limit engagement are pertinent, since ApBM’s effectiveness depends on consistent, repeated engagement with what might feel like an abstract or artificial task.

This study aimed to address the uncertainty around how people accessing AUD treatment perceive ApBM apps as a potential treatment approach and foreground the multiple forms it takes in consumer encounters and treatment contexts. To do this, we took an evidence-making intervention approach, as proposed by Rhodes, Closson [20]. Conventional evidence-based intervention and implementation science discourses “tend to imagine a stable intervention object with predictable effects if implemented correctly” irrespective of the implementation context [20]. As a result, evidence-based intervention discourses assume an intervention’s likely effectiveness can be known prior to its implementation by consulting evidence that may have been generated in other intervention contexts. In contrast, an evidence-making intervention approach focusses on how interventions are ‘made’ through local implementation processes and experiences, comparing the different meanings and effects produced to those intended or outside the local implementation contexts [20]. Rather than suggest that ApBM apps are (or should be) a singular, invariant phenomenon, we aim to draw attention to the multiple ways ApBM apps can emerge in practice.

## Methods

### Study design and context: AAT-app RCTs

Qualitative interviews were conducted with participants with AUD from two RCTs of AAT-App. AAT-App is a later iteration of SWIPE which addressed technical and design issues, and desired features suggested by previous quantitative and qualitative research [12, 18]. One trial recruited people receiving outpatient treatment from publicly-funded addiction treatment services in the Melbourne metropolitan area, and aimed to test whether providing the app as an adjunctive intervention helped them reduce their alcohol use [13]. The other trial recruited clients from five short-term residential rehabilitation services (1 public, 4 private; typically offering 1–3 month programs) in the state of Victoria, Australia, and provided the app to them at the time they discharged from residential treatment, to test whether it helped prevent relapse [14]. The app had consumer input in its design at several stages of development.

In both trials, participants were randomised to receive either a version of the app which delivered ApBM, or a version that delivered a limited amount of sham training (a training task designed to resemble cognitive training, but which was not designed to change approach bias). In the ApBM condition, participants were prompted, after installing the app, to select 6 alcohol-related images that resembled the drinks they tended to consume, and 6 positive images related to their goals, motivations, or healthy activities they would like to engage in more. Participants could select from libraries of images provided in the app, or use images stored on their own phone (camera roll). Selected images were then used in the ApBM sessions.

Participants in the ApBM condition received app notifications reminding them to complete two ApBM sessions per week for 4 weeks, though they could complete more sessions if they chose to, and could continue accessing the ApBM task after the 4-week intervention period if they desired. Each ApBM session took approximately 3–5 min to complete, and involved 156 trials where participants were instructed to push away images (by swiping up on their phone screen, causing the image to shrink and disappear) if they had a portrait-orientation frame, or pull towards themselves (by swiping down on their phone screen, causing the image to expand) if the image had a landscape-orientation frame. Alcohol images had portrait-oriented frames (i.e., push away) 92% of the time, and landscape-oriented frames on 8% of trials. Conversely, positive images were landscape-framed (i.e., pull towards) 92% of the time, and portrait-framed 8% of the time.

To further encourage participant engagement with the app, the ApBM task was gamified with a scoring system, where participants could earn points for each correct response (with faster responses earning more points) and lose points for incorrect responses. At each new session, participants were encouraged to try to improve on previous sessions’ scores. In both projects, quantitative outcome data was collected at follow-ups 4, 8, and 16 weeks after participants installed the app. Further details of each trial’s methodology, including recruitment methods, eligibility criteria, the app, and other quantitative data collection conducted in these trials, are available in the trial registrations [13, 14, 21]. We have also included relevant screenshots of the app in Additional file 1.

### Sampling and recruitment for qualitative study

In both projects, participants were asked, at the end of the 8-week follow-up online survey, to indicate if they would like to be contacted about participating in a qualitative interview. If they indicated interest, they were referred to an unblinded qualitative interviewer, separate from the blinded research staff administering RCT recruitment and quantitative data collection, who identified whether the participant was in the ApBM group. Since we were interested in participant experiences of the ApBM app that could guide implementation of this intervention in clinical practice, we did not invite participants from the sham-control group to participate in qualitative interviews (since sham-control training would not be implemented outside of RCTs). Otherwise, this was a convenience sample, where we included the first 20 participants (10 from the outpatient trial, 10 from the rehabilitation trial) who indicated interest and who we were able to contact and arrange interviews. A sample size of 20 was chosen based on our past experience and research reiterating that similarly-sized samples in qualitative research typically achieved data saturation – the point at which no new themes emerge [22]. We also confirmed this by keeping an audit trail and iteratively monitoring coding and theme development for saturation. We approached 35 RCT participants to participate in qualitative interviews, but 12 did not respond to our contact attempts; 1 declined to participate due to lack of time; 1 made an appointment for an interview but then did not answer their phone or respond to further contact attempts; and 1 interview was terminated early by the interviewer due to perceived hostility. Characteristics of the 20 participants who completed interviews are shown in Table 1. All outpatients were receiving counselling related to their alcohol use, and some were also receiving case management (*n* = 4) or anti-craving medication (*n* = 1). All participants were of White/European ethnicity.

**Table 1:** Participant characteristics

Variable	Whole sample (*N* = 20)	Outpatients (*n* = 10)	Rehabilitation clients (*n* = 10)
**Age**: mean (range)	52.1 (22.5–67.3)	57.1 (36.2–67.3)	47.1 (22.5–66.8)
**Gender**: n (%)			
Female	8 (40)	4 (40)	4 (40)
Male	12 (60)	6 (60)	6 (60)
**Highest education level**: n (%)			
Year 10 or 11	1 (5)	1 (10)	0
Year 12	1 (5)	0	1 (10)
Apprenticeship, TAFE certificate/diploma, or professional qualification	12 (60)	8 (80)	4 (40)
Bachelor degree	4 (20)	1 (10)	3 (30)
Postgraduate degree	2 (10)	0	2 (20)
**Treatment goal**: n (%)			
Stop drinking	16 (80)	6 (60)	10 (100)
Reduce drinking	4 (20)	4 (40)	0
**Baseline AUDIT Score**: mean (range)	27.5 (12–40)	24.1 (12–32)	30.9 (12–40)
**Number of ApBM training sessions completed**: mean (range)	10.0 (6–14)	10.0 (6–14)	9.9 (7–14)

ApBM: Approach bias modification; AUDIT: Alcohol Use Disorder Identification Test

An unblinded qualitative interviewer contacted referred trial participants to briefly explain the purpose and procedures of this qualitative study. If the person was interested in participating, the interview was scheduled and they were sent written information on the qualitative study (they had already received written information and consented to the RCT, but this did not include details of the qualitative study). At the beginning of the interview, the interviewer confirmed that the participant had read and understood the information, and recorded their verbal consent to participate before commencing the interview. For participants in the outpatient trial, these procedures were approved by the St Vincent’s Hospital Melbourne Human Research Ethics Committee (HREC; 268/21) and the Monash University HREC (project number: 31260). For those recruited through the rehabilitation trial, approval was provided by the Eastern Health HREC (E22-007-87504), The Melbourne Clinic Research Ethics Committee (project 351), and the Monash University HREC (35421).

### Data collection

Semi-structured interviews were conducted by phone and recorded between 19 December 2022 and 28 August 2024. Four qualitative interviewers were involved in this project, including author ADR and three other researchers named in the Acknowledgements. Different interview schedules were used for participants from the outpatient (interview schedule written by AR) and rehabilitation trials, reflecting the different contexts of treatment, recruitment, and use of the app, but were broadly similar, and are available in Additional file 2.

Interviewers enquired about the participants’ experience of treatment at the time they were recruited to the RCT; their concerns about their alcohol use at that time; why they were interested in participating in the RCT; positive and negative experiences of using the app; physical and temporal contexts they used the app in; their reason for using the app; perceived effects of the app (e.g., on alcohol craving or use); perceived compatibility between the app and other treatments or supports they were receiving; understanding of the app’s mechanism and purpose; and types of images they selected for the training task. Questions reflected the evidence making intervention approach’s focus on eliciting contextualised and diverse accounts of intervention experiences and knowledges. Interview durations ranged from 11 to 39 min (mean: 23.6). Participants were given a $40 (Australian dollars) supermarket voucher for participating in the interview. Interviews were transcribed by a transcription service.

### Data analysis

Transcripts were checked and de-identified and imported into the NVivo qualitative data management software. Transcripts were analysed for common themes using the framework approach to thematic analysis [23], which can combine aspects of descriptive thematic analysis (e.g., codebooks) and reflexive thematic analysis (e.g., development of meaning- based themes) [24]. This well-established approach, which is particularly suitable for applied or practice-relevant research, was considered appropriate as it enables themes to be derived both deductively, based on the research questions and theory, and inductively based on content identified in the data [23]. After reviewing and familiarising themselves with transcripts, two of the authors (MCLB and JCBP) each initially coded five transcripts and their preliminary coding was reviewed by a third author (MS). Guided by the study’s aims (deductive) and emerging content from initial coding (inductive), these two authors (MCLB and JCBP) further developed the coding framework in discussion with other authors (VM, JBBG). The remaining transcripts were then coded by MCLB and JCBP using this coding framework. Codes were reviewed, merged, refined, and analysed for commonalities and divergence. In discussion between MCLB, JCBP, MS, VM, and JBBG; and with reference to the evidence-making intervention approach, themes were generated and refined through the process of writing up the analysis. VM, JBBG, HP, and DW are trained in psychology and have extensive experience conducting research on the efficacy of ApBM for addiction. Hence they are highly knowledgeable about the quantitative evidence for ApBM’s efficacy, its proposed cognitive mechanisms, and previous qualitative research on ApBM for AUD [18], but these preconceptions may bias which data they were attentive to in the present study. Nevertheless, MCLB and JCBP took primary responsibility for thematic coding of transcripts, with support from ADR and theoretical guidance from MS. MCLB, JCBP, and ADR are trained in psychology, but were relatively less familiar with ApBM, while MS is trained in qualitative sociology and has not been previously involved in ApBM research, and was hence able to provide guidance that was likely less influenced by preconceptions regarding this subject (although his broader research on alcohol and culture may influence his positionality related to this topic). AR was relatively unfamiliar with ApBM when he led the development of the interview schedules, and the interviewers also generally had little past involvement in ApBM research, but all may have been broadly influenced by their training in psychology. DIL is a psychiatrist, leading addiction researcher and director of an addiction treatment, research, and workforce training organisation, and hence primarily applies a lens to the analyses focused on their practical implications for addiction treatment. While the authorship team’s varying academic and clinical backgrounds may result in a mixture of epistemic views and ontological beliefs, they broadly agreed on the pragmatic evidence-making approach used in this manuscript. In this article, we focus on prominent themes related to how participants understood and used the app, which are most relevant to the aims of this study. The evidence-making intervention approach particularly drew our analytical attention to the ways the app materialised in participants accounts of their interaction with it.

## Results

We describe several different, although not mutually exclusive, ways consumers engaged with the app. Our analysis of these draws attention to various individual, social, and contextual elements involved in shaping how the app materialised in particular ways, especially the gendered dimensions of interacting with the app. The latter was identified as particularly prominent.

### Theme 1: Context and structure of app use (part of daily routines)

In contrast to evidence-based intervention discourses where intervention contexts can often be overlooked, or are treated as a variable to be controlled for, an evidence-making intervention approach views context as critical to how interventions emerge in practice. Indeed, many participants described finding it useful to incorporate using the app into part of their daily routines.

#### Time of day

There was variety in participants’ preferences for the location and time of day in which they would regularly use the app. For some participants, the timing of using the app was shaped by when they tended to experience cravings, with some preferring the morning when they weren’t craving substances:

a lot of time I’d wake up and do it in the morning so I’m not really craving as much as I would in the afternoon, so the time of day to do the app is important. (Participant 32, Outpatient, 36 years, Male).

Here the participant frames the intervention as something which requires their full attention. In contrast, and aligning somewhat with the notion of the app as a craving distraction tool (discussed further later), other participants used the app when they were most likely to consume substances or experience cravings:

Primarily for me in the mornings because I was one of those chemically dependent, so you wake up and you drink. Whilst my body is no longer dependent on alcohol, my brain still had that association …, my brain would instantly go, wake up, have a beer. So that’s when I was using the app primarily. – Participant 38, Rehab, 39 years, Female.

For some other participants, experiences of craving didn’t shape when they used the app as much. Instead, their use of the app was more shaped by when they could fit it into to their daily rhythms, as the following quote indicates:

I was usually doing it when I was on my way to work or something like that. I’d do it on the bus… It was usually before work or after work. (Participant 22, Rehab, 23 years, Male).

Others preferred to find a quiet time in the evenings when they were winding down or had some time alone free from distractions:

It was usually in the evenings. I would say it was pretty well always in the evenings… after dinner … in a relaxed atmosphere after dinner (Participant 21, Rehab, 67 years, Male).

Because of my hectic work schedule, it was generally in the evening. On the couch before I went to bed, usually. (Participant 9, Outpatient, 63 years, Female).

Of note, one participant felt:

it’s like my little, quiet space, and I found that was a good thing, to just, okay, just switch off from the outside world a bit, and just go through the app, and it’ll – what would I say? Yeah, just centre yourself a little bit, I suppose. (Participant 26, Outpatient, 61 years, Male).

Here the app becomes a part of times, spaces, and routines of relaxation and self-care.

#### Ongoing and repetitive nature keeping them on track

Many participants appreciated the frequency and repeated nature of the app tasks, suggesting it served as a reminder of the need to continually work towards maintaining positive habits in relation to their drinking:

The fact you had to continually [be] doing it, I think that actually helps … I found once I started doing it, it was like exercise to me where I got into a routine and I was looking forward to it in a way. (Participant 39, Rehab, 55 years, Male).

I guess it’s more of a gradual process. The more you do it the more you are training (Participant 2, Rehab, 37 years, Male).

In these accounts, the app utilised habit as a mechanism to generate effects. Habit is often considered to be implicated in substance use problems. However, habit is also something that substance use treatment disrupts. For instance, one participant noted that having a routine was particularly important after first leaving inpatient treatment:

it made me just check back in to think about rehab, the reasons that I wanted to stop. When you first get out of rehab, you’re still very fragile, and it just brings it all - it’s just a good reminder, I suppose. (Participant 14, Rehab, 49 years, Male).

Another participant even felt the app could be completed more frequently than the recommended two sessions per week to increase accountability and the benefits seen:

It would have to be done a lot more I think, to – like more frequently maybe, going through the app more frequently, because then it just reminds you that you’ve got to do it and it may hold you self-accountable a little bit. That might help, I don’t know. (Participant 10, Outpatient, 65 years, Female).

Consistent with her views here, this participant completed an additional 3 ApBM training sessions to the 8 recommended (i.e., 11 total).

### Theme 2: Gendered responses to gamification elements

#### App as a game (among males)

One of the main ways participants engaged with the app was as a game and this engagement style (which was likely facilitated by the gamification elements deliberately included in the app) was prominent amongst male participants, highlighting how interventions may emerge in gendered ways. Rather than interacting with the app primarily as a therapeutic intervention, one participant exemplified this engagement style saying:

I saw it more as a game to complete. I saw it as like a quest … no negatives. I think it was actually fun to play at some points. (Participant 22, Rehab, 23 years, Male).

The focus and motivation of participants who engaged with the app as a game was on virtual rewards, such as obtaining a high score:

I, yeah, tried to do it as fast as I can. I wanted to get the most points … It’s got the little reward, hey, new high score or whatever, so there’s a little bit of a ‘well done’ so you sort of feel a little bit good about yourself (Participant 2, Rehab, 37 years, Male).

I wanted to continue. I really, I had this compulsion to complete you know? I just wanted to finish – I really wanted to finish the levels. (Participant 22, Rehab, 23 years, Male).

For such participants, prioritising speed and a high score came at the expense of deep engagement with imagery displayed in the task:

I wasn’t even looking at the images by the end of it, I had my hands and my [thumb] on the screen ready to go … It was just, is this a square or is this a rectangle, I’m not even looking at the image, … just something that was shaped and as soon as I see that shape, bang, I’m not concentrating on the image. (Participant 30, Rehab, 47, Male)

The desire to obtain high scores aligned with participants’ self-described competitive motivations and dispositions, and notions of competition as characterising dominant forms of masculinity. Competitively inclined participants tended to describe the app as appealing and engaging:

I guess I was quite competitive, I wanted to get a good score and so it is like a game and the app gives you your score at the end of it and it says well done if you’ve got a new high score. So, that aspect I found really good. It’s engaging and gets people to want to do it. (Participant 2, Rehab, 37 years, Male).

As illustrated in these quotes, achieving new high scores was motivating and exciting for male participants who engaged with the app as a game.

#### Monotony of task (females)

In contrast, female participants tended to report that the app was repetitive and boring:

I probably found it more monotonous that it was just the same thing, up or down (Participant 3, Rehab, 58 years, Female).

it can be a little monotonous. (Participant 38, Rehab, 39 years, Female).

Female participants noted becoming familiar with the task and images they selected after several sessions, and that this contributed to the app being boring:

But, yeah, by the time – by the fourth week, it was like okay, same images, same images, and I almost felt like I could almost second-guess it … by week four, got a bit boring. (Participant 30, Outpatient, 57 years, Female).

A couple of things I found a bit tricky was that because the same images were used all the time, you became very familiar with them. … I also felt that very quickly my brain became very used to seeing all the different images and knowing which one went which way. (Participant 9, Outpatient, 63 years, Female).

These participants also provided suggestions for ways in which the app could be made more engaging by incorporating additional features, which might encourage deeper and more personalised interaction with the app:

The only reward was the high score. Maybe there could be a bit more – I don’t know, I quite like the quotes that come up each day on my ‘I Am Sober’ app, where I pledge, so the pledging, saying you know I’m not going to drink today, and then a quote comes us that you can sort of remember in your head. … So maybe there’s just something a bit more that could go with just to satisfy the reward system. (Participant 30, Outpatient, 57 years, Female).

It didn’t ask me why I was drinking, it didn’t ask me what the triggers were … I mean people have different triggers that trigger them to drink and there was never ever a question about that. … there are different things that trigger me, you see and there are habitual things that do that. (Participant 10, Outpatient, 65 years, Female).

These quotes suggest possible improvements that could make the app more engaging and more responsive to individual’s contexts and motivations, especially for those who don’t engage with the app as a game.

### Theme 3: Multiple perceived ways the app works

In this theme, we elaborate on participants varying perceptions of the ways the app works in practice.

#### App as a craving distraction tool

Participants appeared to hold different perceptions of the app, with some conceiving it as a tool to distract from feelings of cravings (i.e., through repeatedly rejecting/avoiding alcohol images):

It was distracting me from wanting to have any cravings. (Participant 30, Outpatient, 57 years, Female).

Here the app is framed as a brain-training tool to prevent cravings and substance use.

#### App as a recovery reinforcer

In contrast, others regarded it as a tool for reinforcing one’s recovery goals:

It kind of reminded me why I was doing it. You know it reminded me you’re not drinking – don’t drink. (Participant 30, Outpatient, 57 years, Female).

Many participants noted that this was particularly reinforced through the repetition of approaching their positive images, helping them to focus on other aspects of life:

I found that when I was looking at the pictures of the alcohol, it wasn’t actually making me want to drink alcohol, but when I was looking at the positive images that I had chosen, it was making me want to go and participate in those positive experiences. (Participant 33, Rehab, 45 years, Female).

[the app] trains your mind to actually understand that there’s a difference between how much you drink and how much you should enjoy life and the options there. So providing alternatives to actually having a drink by actually using different images that shows you either doing sport or being out and about. (Participant 27, Outpatient, 60 years, Male)

it makes you think, well, no, real life is the priority thing, and drinking is a secondary thing, so to speak. So, in that context, it tries to break that cycle … It sort of helped – the app, or just the whole process … made me think, well there’s other, more important priorities. (Participant 26, Outpatient, 61 years, Male).

Specifically, some participants discussed the motivations or intentions behind the positive images they selected to use within the app, including “pictures that you wanted to take into your new world … motivators to not drink alcohol” (Participant 14, Rehab, 49 years, Male), “what you want from sobriety and your happy life” (Participant 40, Rehab, 53 years, Female), and “my interests that I wanted to do as recreation and also things that I had set in my time in clinic as my goals … what I’ve wanted to be doing instead of drinking” (Participant 33, Rehab, 45 years, Female). Here the perceptions of the app reflect ideas of recovery that circulate in rehabilitation settings in particular, which are not only about changing substance use, but also about cultivating wellbeing.

One participant used breathing as a metaphor for this mechanism:

bringing the good things towards me or filling me up and then pushing the things I don’t like. Kind of like breathing maybe, breathing in the good things and exhaling the negative. (Participant 39, Rehab, 55 years, Male).

The personalisation aspect of the alcohol stimuli in the app was appreciated by some participants:

I liked the idea that you could pick your poison of choice at the beginning of it. I liked that. Obviously, then on the flip side of that was trying to find pictures that you wanted to take into your new world. (Participant 14, Rehab, 49 years, Male).

Like other participants who had been in rehabilitation, the app was potentially entangled in their recovery journeys and the creation of new recovery social worlds, which participants contrasted with their old substance use worlds and practices. Responding to the contextually-situated nature of their substance use, some participants noted that personalisation of the training could be enhanced if, in addition to selecting the alcohol images they were trained to avoid, they could also select “contexts” in which the images were presented:

I suppose it could be expanded image-wise … You could expand it to different settings instead of just having a photo of a stubbie or a photo of a cask… you could put in a more social situation where when you walk in the room, what’s the first thing you see? Like what I do, I just spot these drinking situations, who’s drinking or is there alcohol. (Participant 15, Outpatient, 67 years, Male).

Echoing the importance of context mentioned earlier, this quote indicates a socially and contextually attuned understanding of substance use and triggers, which the images available in the app might overlook. Reiterating the gendered dimensions of using the app, participants also thought it would be useful to have more gender and other diversity in the images included in the app library for participants to select from for training, to ensure that the app resonates with various groups of people:

The only thing I would add, and this is just me personally, is that the only exercise picture I could find in there was a man lifting weights. It was supposed to be a positive picture for me, but yet it weren’t because I don’t lift weights. I want to go out running or I want to go swimming. … I just remember thinking why would that be the only exercise-related picture you have when exercise is fundamentally important to help keep people sober and keep their mental health up. I think for me, I would have preferred to have more options of what exercise might look like for different people. It is not necessarily just a man lifting weights. (Participant 33, Rehab, 45 years, Female).

#### App to counter environmental triggers

Participants noted the prevalence of alcohol-related stimuli they are exposed to within their environment, which impacted their recovery. Some felt that the purpose of the app was to help desensitise them to these images of alcohol, which are almost unavoidable in day-to-day life:

it’s meant to train your brain to react differently to triggers, visual triggers, that you may come across when out in public or watching TV or something (Participant 32, Outpatient, 36 years, Male).

I suppose it tries to train your brain to actually see the image as a trigger not to drink. (Participant 27, Outpatient, 60 years, Male).

#### Subconscious mechanisms

Some participants, particularly those who were unsure about the mechanism behind the app, speculated that the app may have been acting on their thoughts and behaviours subconsciously:

…maybe subconsciously something was going on. On the surface level there was not much happening. (Participant 22, Rehab, 23 years, Male).

I would imagine that it’s about training the subconscious to be averse to the acceptance of alcohol (Participant 21, Rehab, 67 years, Male).

They also described the importance of the app tapping into the automatic biases that many people who are trying to reduce their consumption experience:

I feel like it’s supposed to be done quickly. It’s supposed to be very active, see and react straight away, because I guess that’s how in my experience it’s sort of worked in the past. When I have been drinking, I can be sober and out and about in public and I just see it. I have no intention of having a drink, but I see a bottle shop and my brain just, you know, the neurons in my brain just go bang, like just go straight in there right now and pick up a bottle of wine for $9. I guess it’s sort of - the idea of the app is to combat that spontaneity I think, but that was my experience. (Participant 2, Rehab, 37 years, Male).

As is evident in these quotes, participants who saw the app as acting on the subconscious also tended to invoke neurobiological explanations of substance use and recovery – focussing on the brain and its automatic processes.

### Theme 4: App as complementary to treatment

Rather than a stand-alone intervention, most participants felt AAT-App would be best used in conjunction with other addiction treatments and supports, and that it complemented these well. Participants intentions for using the app often aligned to seeing its usefulness as an additive intervention, and the idea of the app as a tool in a recovery toolbox:

it [the app] probably needs to be used in conjunction, you know, with counselling and stuff. … Not as a standalone something. (Participant 23, Outpatient, 55 years, Male).

a good secondary thing to do, as well as counselling and stuff. It’s good all round, is all I can say. (Participant 26, Outpatient, 61 years, Male).

it [the app] was not inconsistent in any way, with other supports that I had. I felt it complementary. One facet of … a multi-faceted, a multi-dimensional approach … a tool in a suite or a toolbox of – yeah – the methods to achieve the end that you want to achieve (Participant 21, Rehab, 67 years, Male).

One participant felt using the app in between counselling sessions helped to keep them on track with their drinking behaviours:

doing the app in between is good because it’s a bit of – it seems to me like a bit of assistance, and you’re thinking about these things more regularly in between going and seeing the counselling people. So, it keeps you thinking about habits, and drinking excessively and stuff, in between. (Participant 26, Outpatient, 61 years, Male).

Another participant suggested future implementation of the app could incorporate giving their counsellor access to the app data to discuss during sessions:

they could get hold of some of the data, you know, like, if my counsellor could, you know, that’s more of a discussion point that could be held… I mean that gives the counsellor something to look at and then talk about.” (Participant 23, Outpatient, 55 years, Male).

The accounts in this theme draw attention to the way in which interventions – particularly when thought of as tools – are, and can be better, situated within treatment systems and recovery ecologies.

## Discussion

### Findings and interpretation

This study is the first qualitative exploration of experiences of an ApBM app among people in treatment for AUD. By taking an evidence-making intervention approach, the article highlights diverse understandings and (some unexpected) ways the app is used when implemented in AUD treatment contexts. The first theme was similar to findings by Bolt, Piercy [18] in non-treatment-seekers with hazardous/harmful drinking, whereby many participants incorporated the app into their daily schedules, predominantly in the morning or evening. This mirrors findings by Neale & Bowen [25] that some participants would use their recovery-focused app in the evenings to reflect on their day, while others used it at various times when they anticipated thinking about substance use, felt concerned about their use, or expected cravings to arise. Participants in the current study also recognised how its frequent and repetitive nature could serve to reinforce positive changes, likening it to building a routine or positive habit. Prior et al. [17] similarly found that participants felt the repeated nature of training sessions was important for strengthening changes in alcohol use. In this regard, the app functioned not only as a tool to counter or modify drinking habits, as expected, but also worked to generate new recovery-oriented habits.

Indeed, participants interpreted and used ApBM in ways that went beyond the primary intentions of the clinical researchers who developed it. A key illustration of this in the second theme, related to engaging with the app as a game. Based on the literature on gamification of health apps [26, 27], it was anticipated that the gamification elements of the app would enhance the attractiveness the app, and engagement with its therapeutic aims, especially due to the otherwise repetitive nature of the ApBM task. However, in comparison to most female participants, some male participants were so highly engaged with gamification elements that scoring, speed, and competing with oneself potentially overshadowed engagement with the apps’ therapeutic purpose and other potentially therapeutic functions (e.g., as a reinforcer of recovery-oriented motivations). This finding is consistent with studies of gamified health apps generally, which tend to find that men are more motivated by challenge and competition features than women [28–30]. In part, this may be explained by the observation that such gamification elements align with traditional masculine ideals, such as competitiveness and technical competence [31].

In contrast, female participants tended to comment on its repetitive nature, sometimes finding this boring, a finding observed in other alcohol mHealth intervention research [17, 32]. This suggests that allowing participants to personalise the training task by selecting personally meaningful approach and avoid images was insufficient to overcome the task’s monotony for many participants. Future smartphone ApBM development efforts should explore other gamification and presentation elements, such as use of animations, sounds, feedback, variation in approach and avoid task instructions, and adaptive difficulty algorithms to tailor the task to individual performance (as recently used in an ApBM app for methamphetamine use [33]). Personalisation of the training could be enhanced further by enabling participants to select “contexts” in which images are displayed, consistent with suggestions by researchers who have advocated “ABC” training, a variation of ApBM in which presentation of “antecedent” situations, contexts, or feelings frames the approach/avoidance training trials [34]. In addition, given participants’ reports of boredom and habituation to self-selected images, future ApBM apps should enable periodic changes in the approach and avoid images used. Nevertheless, the repetition and monotony are somewhat unavoidable given the nature of ApBM and its intended purpose to reverse supposedly deeply ingrained action tendencies to alcohol cues. Rather than solely focusing on improving the acceptability of the task itself, future ApBM apps might consider incorporating other recovery-reinforcing features such as goal tracking, peer support, social connection, and self-help content to provide users with additional reasons for engagement [25, 35, 36].

The third theme identified several perceived mechanisms of the app’s effects. The perception of some participants of the app as a craving management tool to counter environmental triggers echoed similar findings by Bolt et al. [18] among non-treatment-seekers. Similarly, participants in the current study suggested the positive ‘approach’ images reinforced recovery goals, while participants in Bolt et al.’s [18] study reported how the positive images served as reminders of what alcohol use takes away. Some participants also interpreted the app’s mechanisms in ways that were consistent with formal cognitive psychology theories of its effects (i.e., as operating on relatively subconscious, automatic responses to alcohol-related cues that people often encounter in their environment). The current study’s participants being a treatment-seeking sample offered unique insights, in the fourth theme, into its adjunctive, complementary relationship with conventional treatment approaches, recognising it as *a tool in a toolbox*, rather than a stand-alone treatment, similar to findings in previous research [25, 37].

Utilising an evidence-making intervention approach has reiterated that, like most health interventions, the app, rather than being a singular tool or intervention, becomes different tools depending on the implementation context and how people engage with it [38]. This reinforces that ongoing research to monitor interventions as they are implemented in different contexts is important, not only to mitigate against undesirable effects, but also to encourage diverse forms of engagement in line with peoples’ needs and desires. Another implication of these findings is that careful consideration of gendered engagement styles and experiences of health apps is needed to ensure apps firstly, contain features that appeal to different genders depending on the target population of the app; secondly, have an adequate balance between gamification and therapeutic features; and thirdly, avoid bias towards inadvertently engaging masculine ideals that may not be helpful or appealing to all app consumers.

### Limitations

Whilst qualitative methods never seek to be representative, a limitation of the study is that the findings may be biased towards participants who had more positive experiences of AAT-App. Participants were recruited only from RCT participants who completed the RCT 8-week follow-ups, and who agreed at that point to be contacted about the qualitative interview. Participants with more negative experiences of the app may have been more likely to drop out of the study prior to completing the 8-week follow-up and/or be less enthusiastic about volunteering to speak about it with a qualitative researcher. This potential for bias is reinforced by the fact that qualitative interview participants had completed a mean of 10 training sessions (when only 8 were recommended), while the mean number of completed sessions among RCT participants more generally was 8. Hence, qualitative interview participants tended to be drawn from those who, for whatever reason(s), were more enthusiastic or willing to complete ApBM sessions than the average participants in the RCTs that this sample was drawn from. However, this does mean they likely had sufficient experience of it to be able to provide meaningful insights. Nevertheless, it is possible that social desirability perceptions may have further biased their responses towards more positive accounts (e.g., if any participants perceived the interviewer as desiring positive comments about the app, or believed that positive comments would be helpful to the researchers).

Typical of samples accessing treatment, participants tended to have relatively high AUDIT scores, and 80% were aiming to achieve abstinence from alcohol. It is therefore unclear if these findings are generalisable to people with milder AUD and/or people who are not seeking treatment, as these populations typically have higher proportions of people aiming for reduced use, rather than complete abstinence; and lower AUDIT scores. The lack of racial diversity of the sample (entirely White/European) is also a significant limitation to the generalisability of our findings. Moreover, although we recruited participants receiving both inpatient and outpatient treatment from both public and private services, which may broaden this study’s generalisability, the transferability of our findings to populations outside Australia remains unclear due to potential differences in treatment systems and other cultural differences between countries.

## Conclusions

This study sought to explore AUD patients’ experiences of smartphone-delivered ApBM to generate insights for improving engagement and efficacy in future ApBM apps. The findings offer nuanced insights into how patients use ApBM and its diverse meanings and effects. People in AUD treatment reported broadly positive experiences with integrating AAT-App into their daily routines and complementing other treatment they were receiving. However, gender differences in engagement were evident. Males tended to approach it as a competitive game, which enhanced their motivation to engage in the training task, but possibly reduced their attentiveness to its intended therapeutic functions; while women tended to find it repetitive and less engaging, but viewed it more as therapeutic tool. Overall, AAT-App was seen as a helpful tool for managing cravings, retraining automatic/subconscious biases, and complementing concurrent treatments (e.g., counselling), with potential to be integrated into clinical care to increase engagement and effectiveness in reducing harmful alcohol use. The findings also highlight the importance of end-user engagement in developing future ApBM apps in accordance with co-design principles. Future quantitative studies should aim to assess how metrics of user experiences of ApBM apps (e.g., acceptability, perceived efficacy, boredom) predict actual app engagement and outcomes, and whether the ways in people engage with an ApBM app (e.g., as a competitive game vs. a monotonous ‘chore’) moderate its effectiveness.

## Supplementary Information

Below is the link to the electronic supplementary material.

Supplementary Material 1

Supplementary Material 2

## Data Availability

The datasets generated and analysed during the current study are not publicly available due to conditions of ethical approval regarding confidentiality. However, data may be made available to specific researchers upon reasonable request to the corresponding author, subject to further ethical approval.

## Abbreviations

AAT-App: Alcohol Avoidance Training App
ApBM: Approach Bias Modification
AUD: Alcohol Use Disorder
AUDIT: Alcohol Use Disorders Identification Test
HREC: Human Research Ethics Committee
RCT: Randomised Controlled Trial
TAFE: Technical and Further Education
