# Supplementary material for: “A tool in a toolbox”: patient engagement with a gamified and personalised approach bias modification app to reduce harmful alcohol consumption – a qualitative study
Source: Addict Sci Clin Pract. 2026 Jan 31;21:22. doi: 10.1186/s13722-026-00646-6 (PMC12874785; doi:10.1186/s13722-026-00646-6)
Supplement: Supplementary file 1 — Supplementary Material 1 [file 13722_2026_646_MOESM1_ESM.docx]

**Inpatient qualitative interview schedule**

**SECTION A: INTRODUCTION, SCREENING, AND CONSENT**

***Introduction***

Thanks for agreeing to be involved in the project we are conducting about your experiences using “AAT-App”.

This interview is expected to take about 30 to 45 minutes. I'll ask a number of questions about your experiences using the app and what you thought of it, but the interview is meant to be pretty informal, and you can consider it like a conversation. There is also an optional mapping task where you are invited to visualise and draw how the app may have influenced your thoughts and behaviours. I’ll explain more about that during the interview. The interview will be recorded. At the end of the interview, I’ll send you a $40 eVoucher for participating. If you would like to take a break just let me know and we can take one part-way through the interview.

Before we begin, could I please confirm that you've had a chance to read and understand the information about the study in the online survey that you completed?

No (either read the PICF to the participant or arrange to call back another time)

Yes.

Do you have any questions before we go on?

***Screening***

We check in with everyone before we start the interview, so I just wanted to know whether you are feeling up to conducting the interview today?

No (direct to Counselling Online/Directline details listed on PICF if participant indicates distress)

Yes.

[**IF NO**] Would you like to conduct the interview at another time?

No

Yes, arrange another interview time.

***Consent***

Ok, so before we start the interview, I will turn on the recorder and begin by recording your consent to participate. Is that ok with you if I start recording now?

**RECORDER ON**

I’ll now record your consent to participate in the interview. Can you please state your full name first. [participant states name]

I will now read out a list of questions, if you could answer either ‘yes’ or ‘no’ at the end of each.

- I have read the Participant Information Sheet, or someone has read it to me, and I understand the purposes, procedures, and risks of the research. No Yes
- I have had an opportunity to ask questions and I am satisfied with the answers I have received. No Yes
- I freely agree to participate in this research project as described and understand that I am free to withdraw at any time during the interview. No Yes
- I give consent for the interview to be audio-recorded. No Yes

I also need to confirm some details about the mobile phone number that you used for this study.

- Is the mobile number I’m phoning you on the same as the phone number you originally used to sign up to AAT-App?
- [**if no to previous question**] What was your old mobile number (the one you originally used when signing up to the study)?

**RECORDER OFF**

**RECORDER ON**

**SECTION B: INTERVIEW**

***[Main dot-points represent the primary interview questions, sub-dot-points represent suggested prompts or follow-up questions that may be used as appropriate]***

- To begin with, can I please ask what were the circumstances that led to you going into rehab?
  - Social circumstances (e.g. employment, social relationships, housing)
  - Mental and physical health/wellbeing
  - Alcohol and other drug use/history/concerns
- In regards, to your rehab admission:
  - How long were you in rehab for?
  - Can you tell me about a typical day in rehab?
  - What treatment and/or supports did you have organised when you left rehab?
  - What made you want to use the app? (e.g., trial; remuneration, address drinking)
- We are interested in your experiences of the app.
  - What were your expectations of the app (if any)?
    - Were those expectations met?
  - How did you find using the app?
    - - Flexibility / ease of access?
      - Technical concerns? (e.g., bugs/glitches)
      - Design (gamification/games/scores)
      - Feedback from the app
      - Effects on thoughts/behaviours? (e.g., circuit breaker/substitute app for drinking/desensitize)
      - Other negatives? (e.g., triggers drinking)
- How and when did you use the app?
  - What times and places did you use it?
  - Can you tell me about a typical time when you used the app? What were you doing and where were you? Talk me through a specific experience if you like.
- Can you tell me about any effects that the app had on your alcohol cravings or drinking?
  - Cravings and triggers?
  - Consumption (e.g., days drinking; quantity)?
- How does the app fit in with any other supports you have (e.g., family, friends, peer support, psychology)?
- Interested in your thoughts about how the app works. <*invite participant to complete the Brain Mapping task>*
  - What’s your view on how the app works?
    - It’s sometimes described as a form of “brain training” – what’s your experience of that like?
  - How would you explain the app to another person if you described it to them?
  - What do you think about the information provided in the app about how the brain-training works?
    - Could the information be improved?
- What images did you choose to use for the app?
  - Did you use your own photos, or the ones provided in the app?
  - Why did you choose those images? What did the images mean to you?
- Do you have any suggestions for how the app could be improved?
  - - Technical issues? (e.g., bugs/glitches)
    - Design (gamification/games/scores)
    - Training / information for future clients
- Would you recommend the app to other people?
  - Would the app be better for some people compared with others?
- Do you have any other thoughts or opinions about the app that you would like to share?

**Conclusion**

Thanks, we’ve covered a lot today and I appreciate you sharing your thoughts about your experiences with the app. We really value hearing people’s feedback, so your input is a huge help!

**RECORDER OFF**

**End of interview wellbeing check:**

Finally, after everything we’ve discussed, I just wanted to make sure that you’re ok and don’t have any concerns by anything we’ve talked about today?

No concerns

Requires support – proceed to support option (see Lifeline, DirectLine, and Counselling Online details on participant information sheet)

**Outpatient qualitative interview schedule**

**SECTION A: INTRODUCTION, SCREENING, AND CONSENT**

***Introduction***

Thanks for agreeing to be involved in the project we are conducting about your experiences using “AAT-App”.

This interview is expected to take about 30 minutes. I'll ask a number of questions about your experiences using the app and what you thought of it, but the interview is meant to be pretty informal, and you can consider it like a conversation. The interview will be recorded. At the end of the interview, I’ll send you a $40 eVoucher for participating. If you would like to take a break just let me know and we can take one part-way through the interview.

Before we begin, could I please confirm that you've had a chance to read and understand the information about the study in the online survey that you completed?

No (either read the PICF to the participant or arrange to call back another time)

Yes.

Do you have any questions before we go on?

***Screening***

We check in with everyone before we start the interview, so I just wanted to know whether you are feeling up to conducting the interview today?

No (direct to Counselling Online/Directline details listed on PICF if participant indicates distress)

Yes.

[**IF NO**] Would you like to conduct the interview at another time?

No

Yes, arrange another interview time.

***Consent***

Ok, so before we start the interview, I will need to turn on the recorder and begin by recording your consent to participate. Is that ok with you if I start recording now?

**RECORDER ON**

I’ll now record your consent to participate in the interview. Can you please state your full name first. [participant states name]

I will now read out a list of questions, if you could answer either ‘yes’ or ‘no’ at the end of each.

- I have read the Participant Information Sheet, or someone has read it to me, and I understand the purposes, procedures, and risks of the research. No Yes
- I have had an opportunity to ask questions and I am satisfied with the answers I have received. No Yes
- I freely agree to participate in this research project as described and understand that I am free to withdraw at any time during the interview. No Yes
- I give consent for the interview to be audio-recorded. No Yes

I also need to confirm some details about the mobile phone number that you used for this study.

- Is the mobile number I’m phoning you on the same as the phone number you originally used to sign up to AAT-App?
- [**if no to previous question**] What was your old mobile number (the one you originally used when signing up to the study)?

**RECORDER OFF**

**RECORDER ON**

**SECTION B: INTERVIEW**

***[Main dot-points represent the primary interview questions, sub-dot-points represent suggested prompts or follow-up questions that may be used as appropriate]***

- To start off, I’m interested in knowing about what kind of treatment you were getting for alcohol use when you decided to try AAT-App. Can you tell me about that?
  - How long had you been involved in that treatment?
  - How were those treatments going for you?
- And what made you want to try AAT-App?
  - Can you describe the concerns you had about your alcohol use when you decided to use AAT-App?
  - Can you describe what was going on in your treatment when you decided to try AAT-App?
- Tell me about your experience using AAT-App. How’d you find it?
  - What were the positives/negatives?
  - Any bugs or glitches that came up?
- How (or in what contexts) did you use the brain-training app?
  - Did you use it at any particular times or places?
  - Did you try using it for immediate craving relief/support when urges to drink arose and, if so, how did that go?
- Can you tell me about any effects that AAT-App had on your alcohol cravings or drinking?
  - [*If they felt it was helpful*] When did you notice it helping?
- Do you have any thoughts about how AAT-App combined with the treatment you were receiving?
- What was your understanding of how the brain-training is supposed to work?
  - What do you think about the information provided in the app about how the brain-training works (if you can remember it)?
  - What influence, if any, do you think that knowing the theory behind the brain-training has on how the app worked (or didn’t work) for you?
- What images did you choose to use for the brain-training?
  - [prompt for information regarding both positive and alcohol images if they only describe one category]
  - Did you use your own photos, or the ones provided in the app?
  - [*If they used photos provided in the app*] What did you think of the photo options provided in the app? (Do you have any suggestions about changes to the options?)
  - What guided your decision about which photos to use?
- Do you have any suggestions for how the app could be improved?
- Do you have any other thoughts or opinions about the app that you would like to share?

**Conclusion**

Thanks, we’ve covered a lot today and I appreciate you sharing your thoughts about your experiences with the app. We really value hearing people’s feedback, so your input is a huge help!

**RECORDER OFF**

**End of interview wellbeing check:**

Finally, after everything we’ve discussed, I just wanted to make sure that you’re ok and don’t have any concerns by anything we’ve talked about today?

No concerns

Requires support – proceed to support option (see Lifeline, DirectLine, and Counselling Online details on participant information sheet)
