# Supplementary figures and images for: “A tool in a toolbox”: patient engagement with a gamified and personalised approach bias modification app to reduce harmful alcohol consumption – a qualitative study
Source: Addict Sci Clin Pract. 2026 Jan 31;21:22. doi: 10.1186/s13722-026-00646-6 (PMC12874785; doi:10.1186/s13722-026-00646-6)

**AAT-App Screenshots (active condition only)**


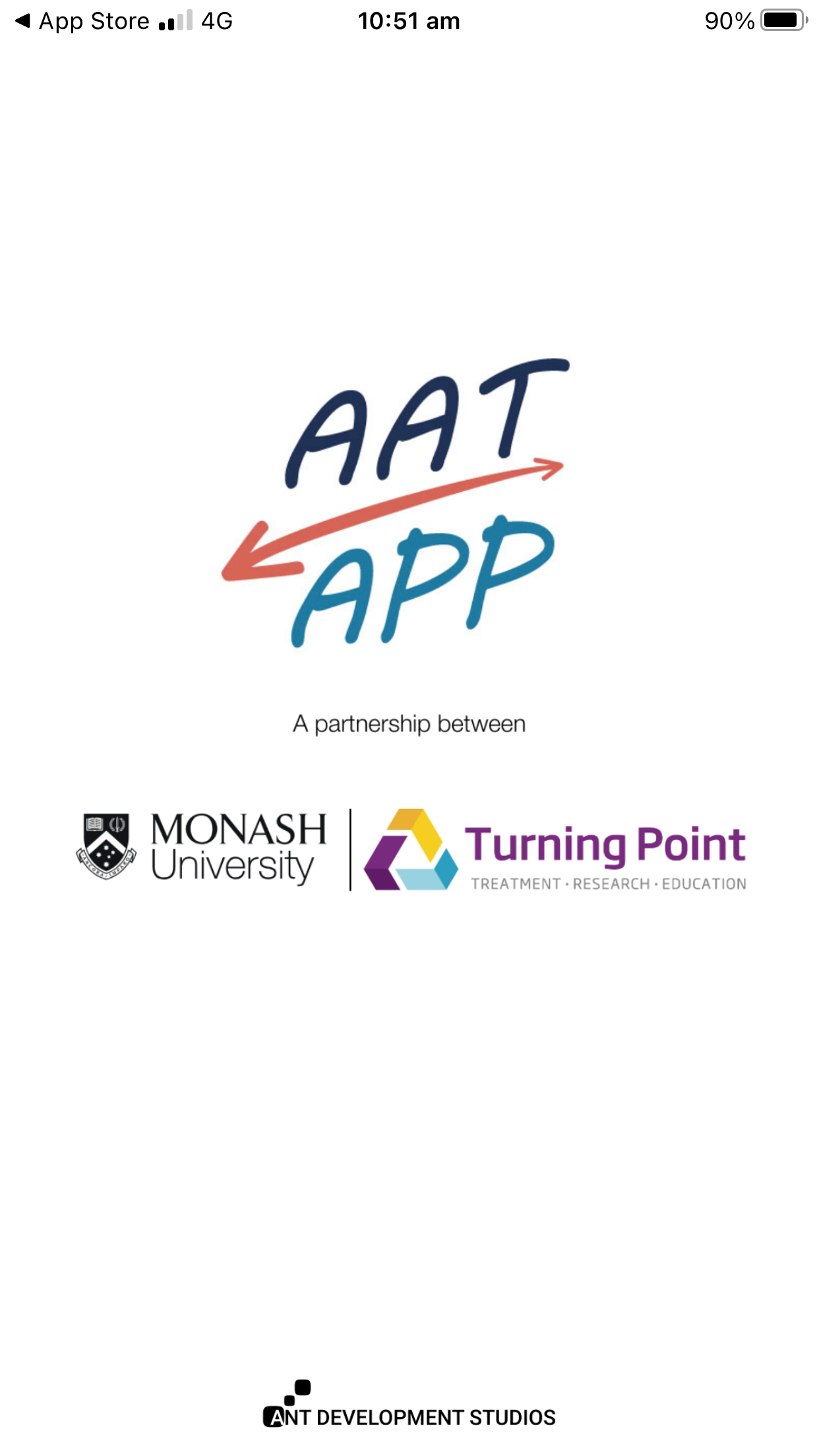

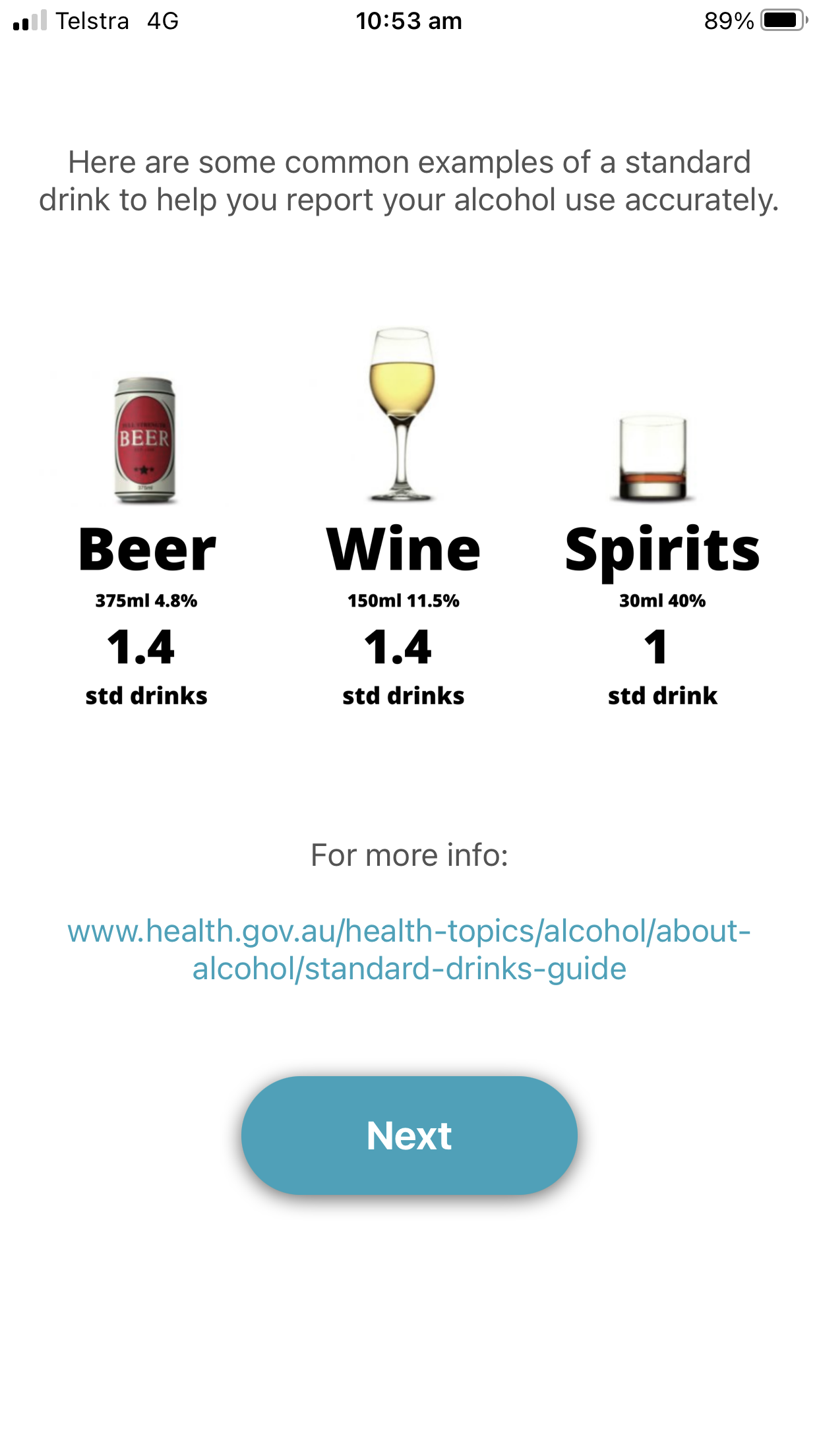


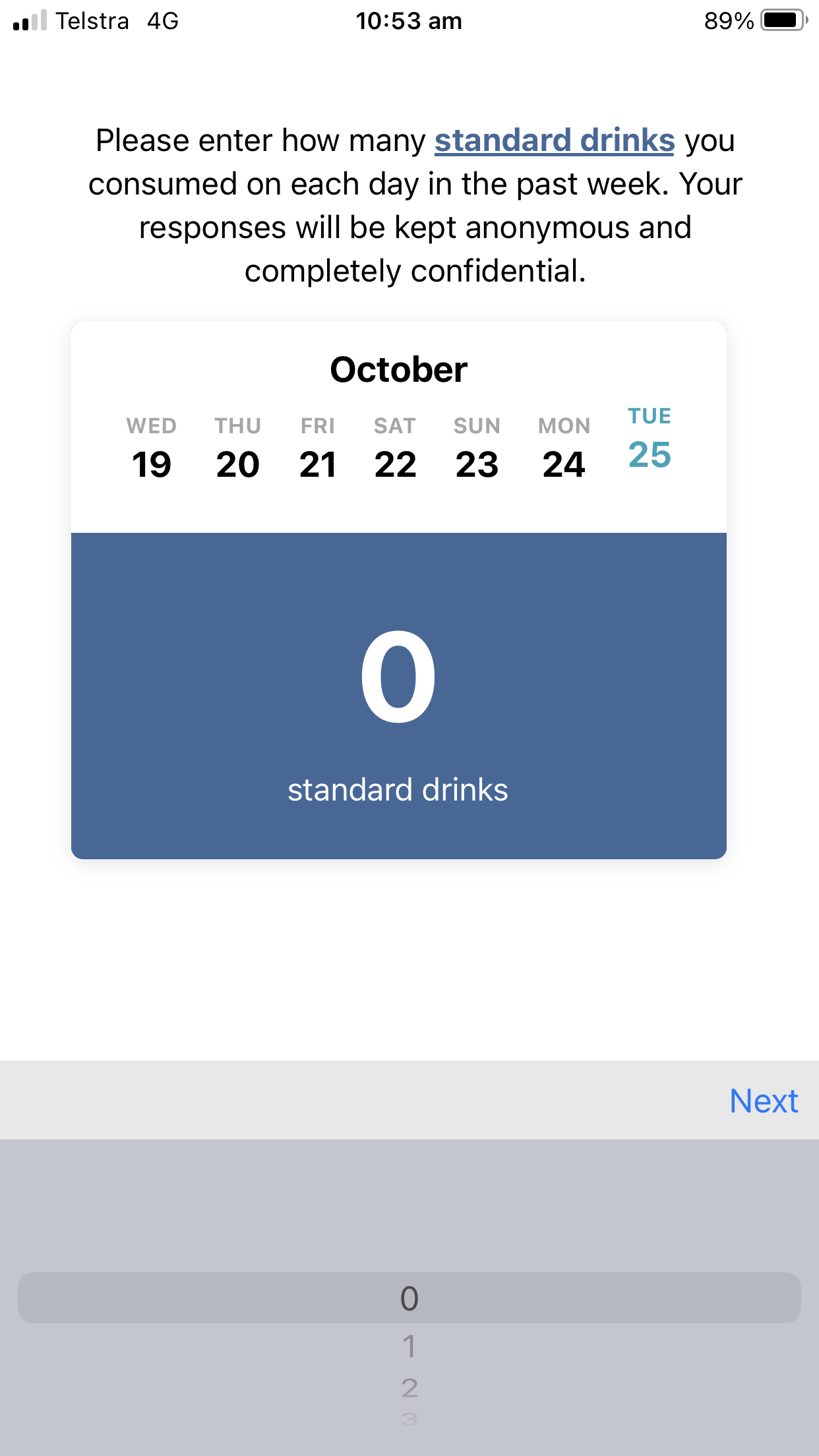

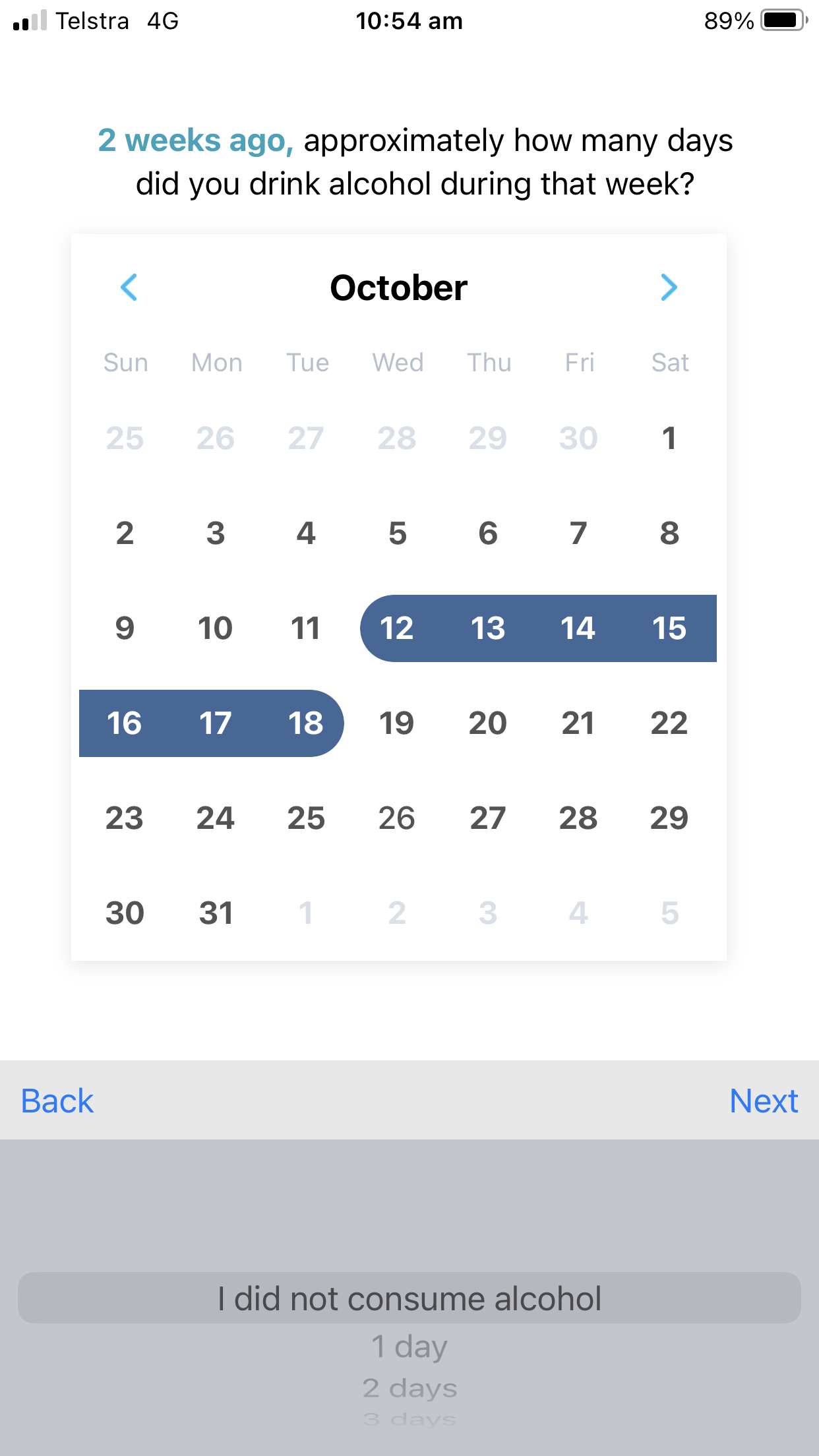


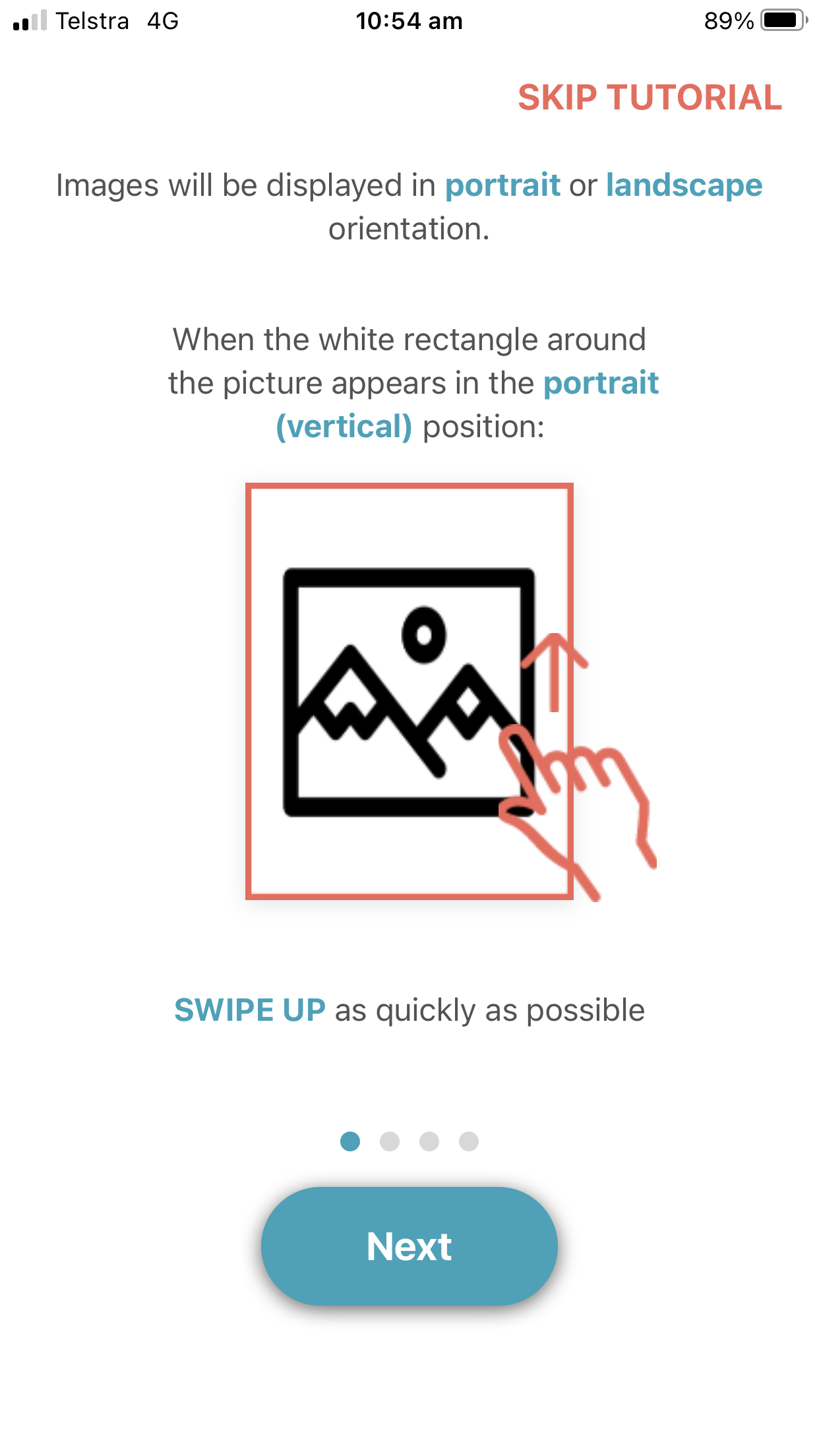

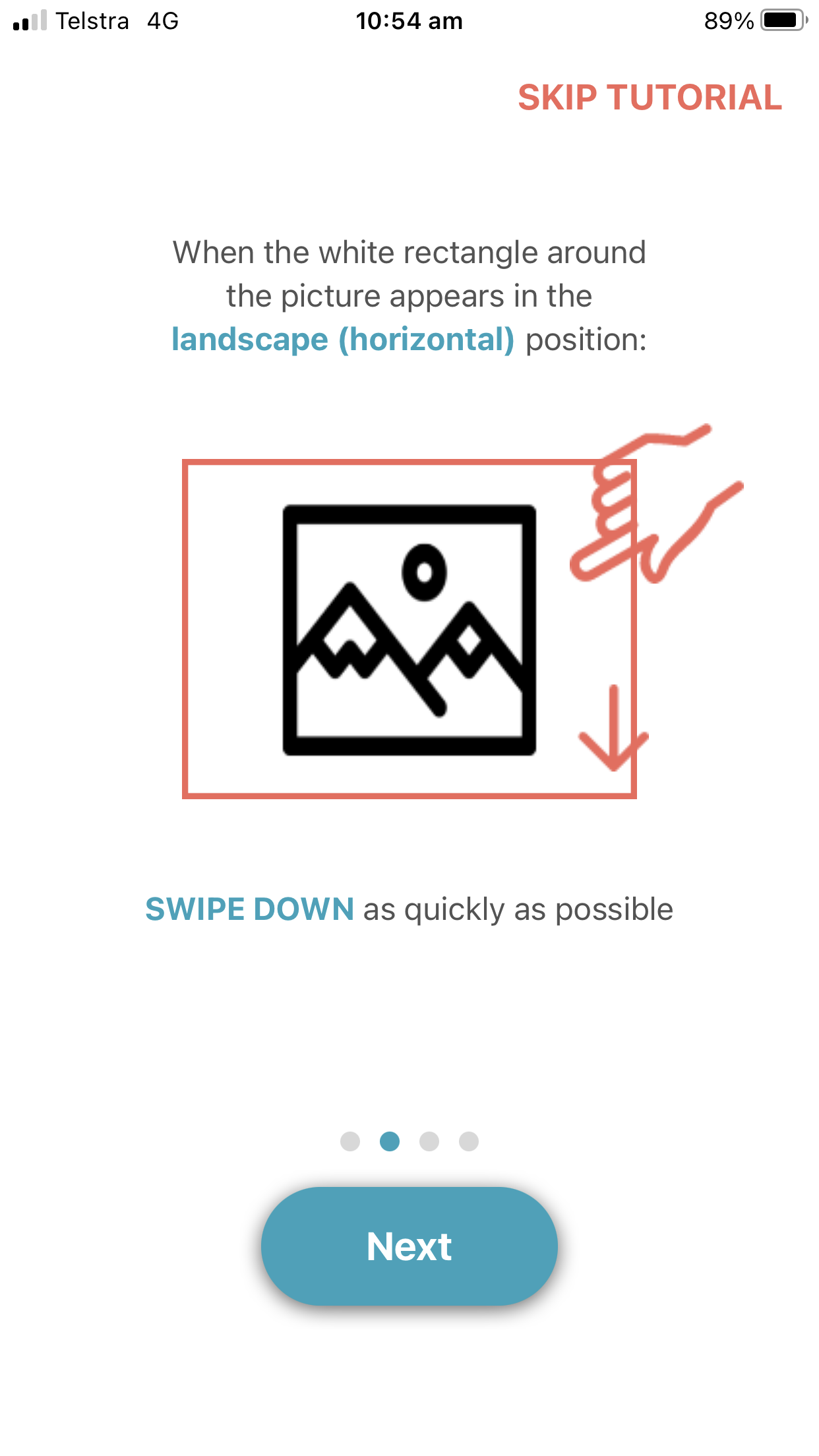


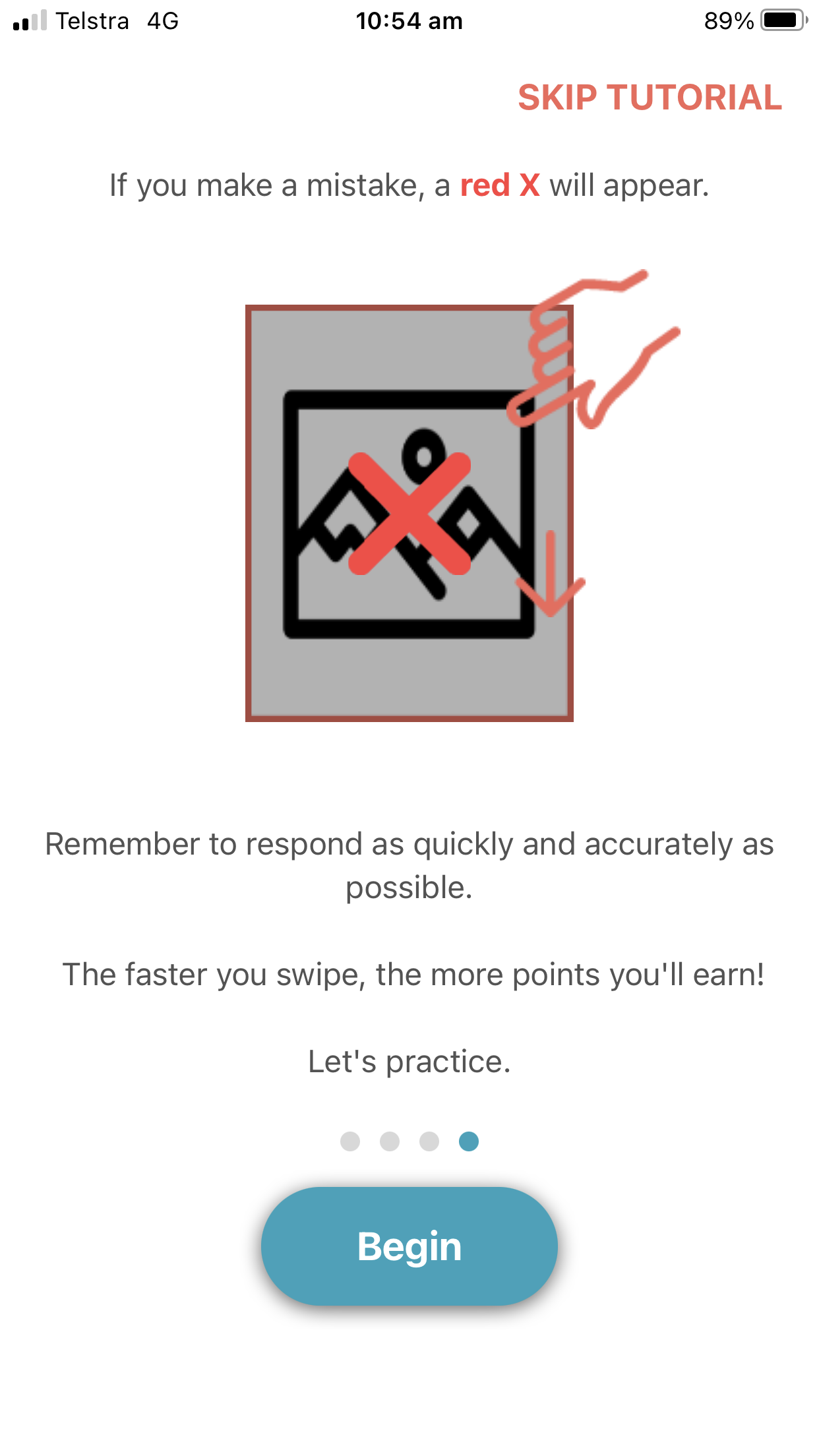

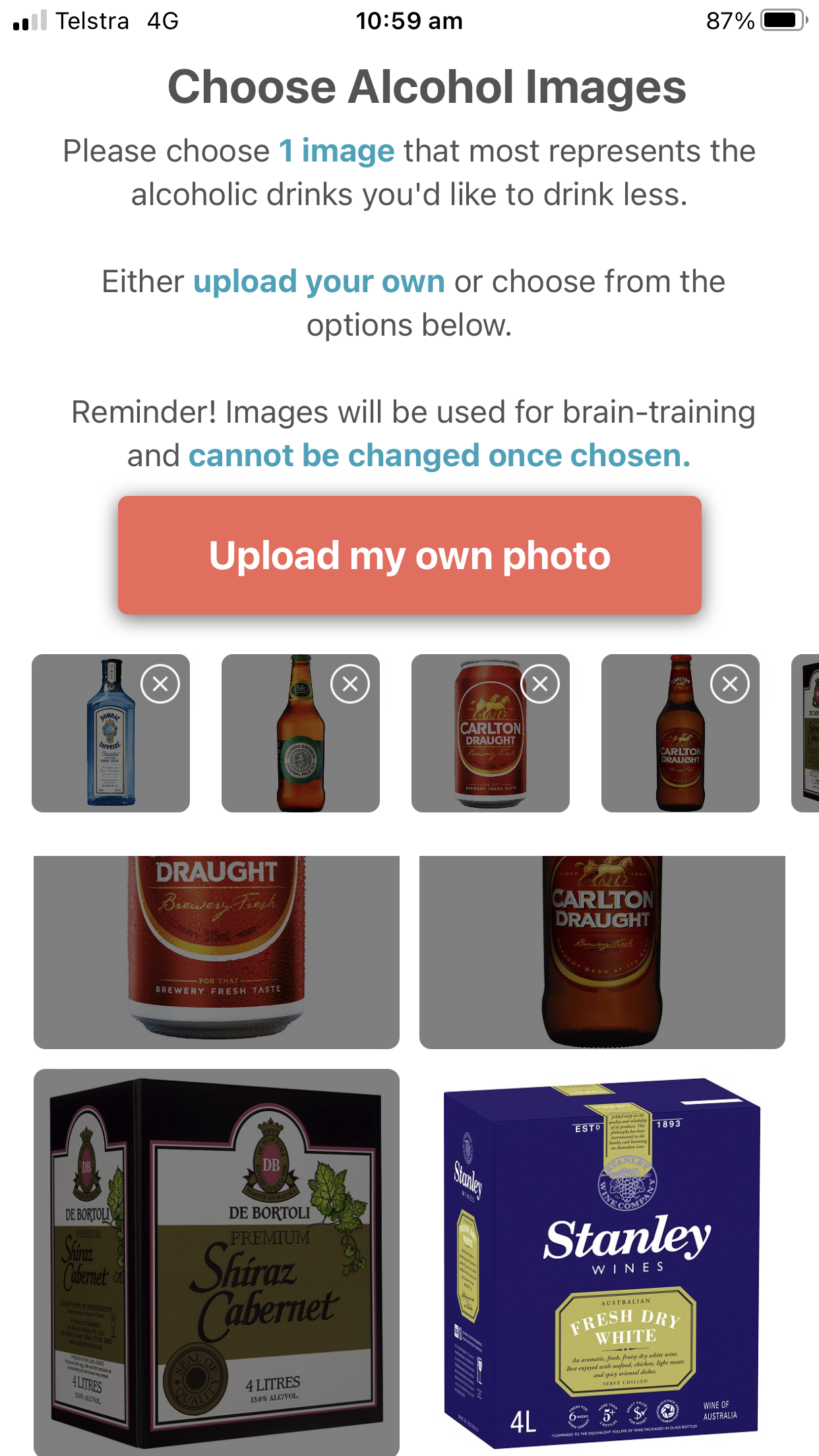


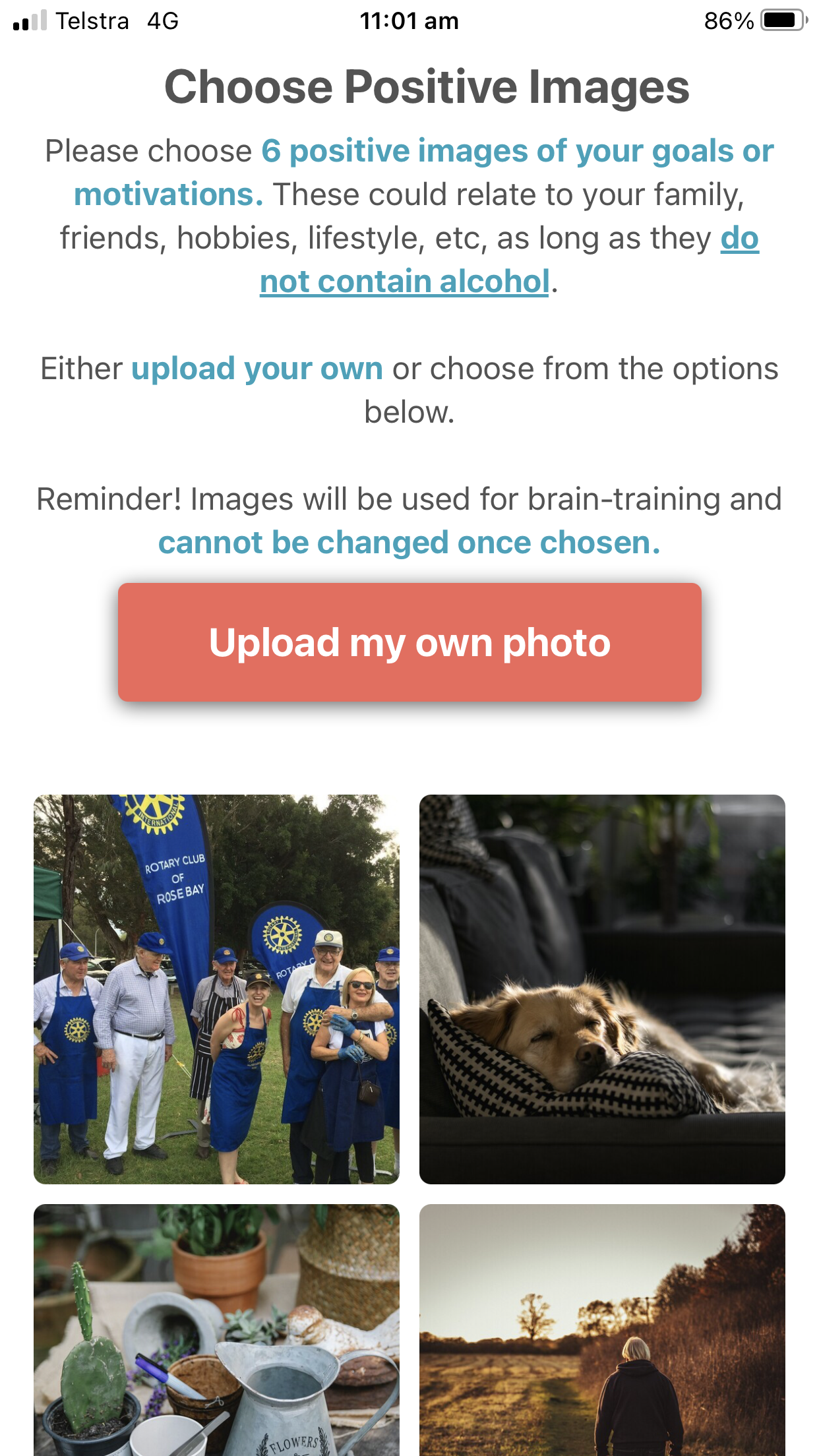

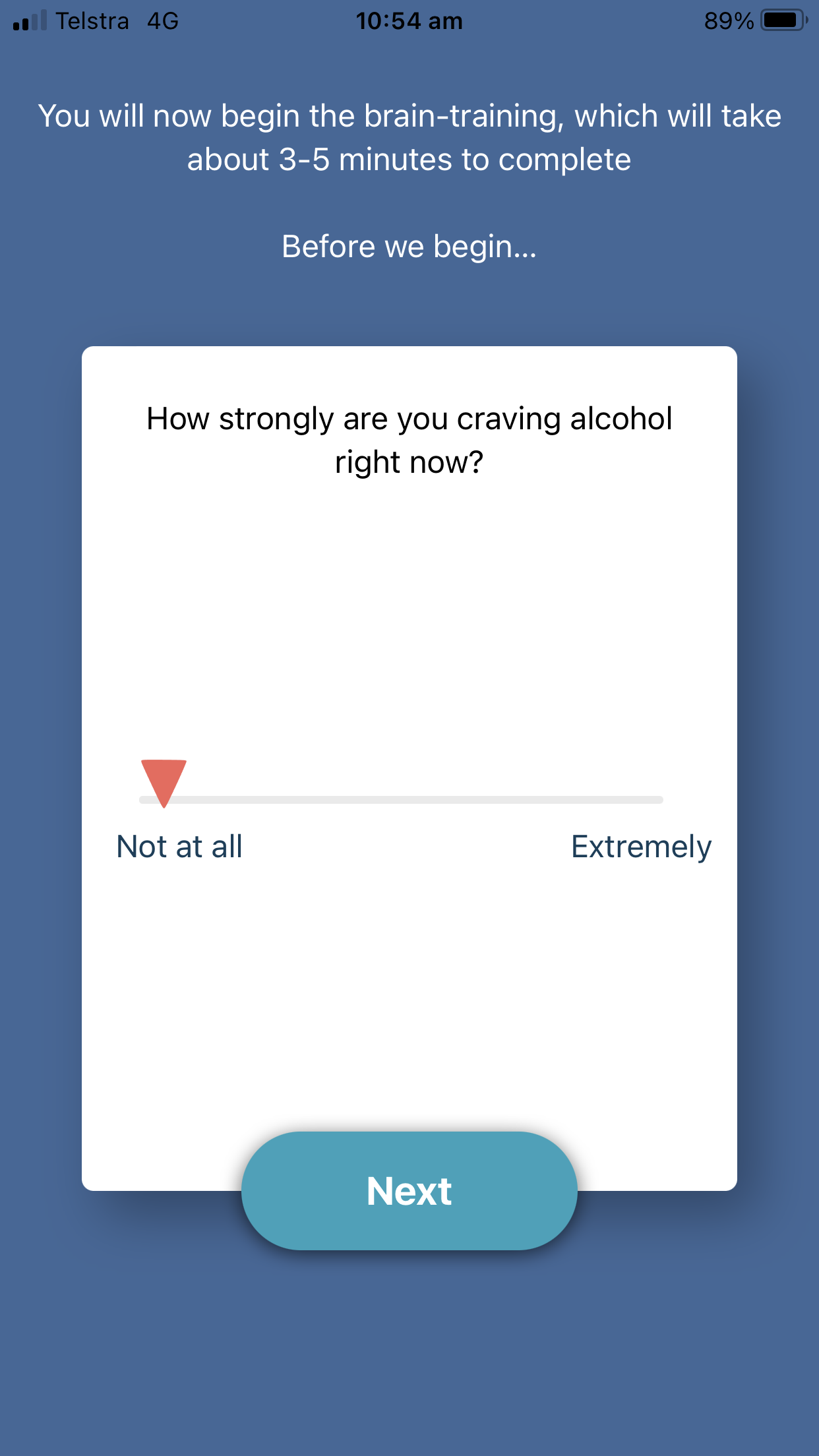


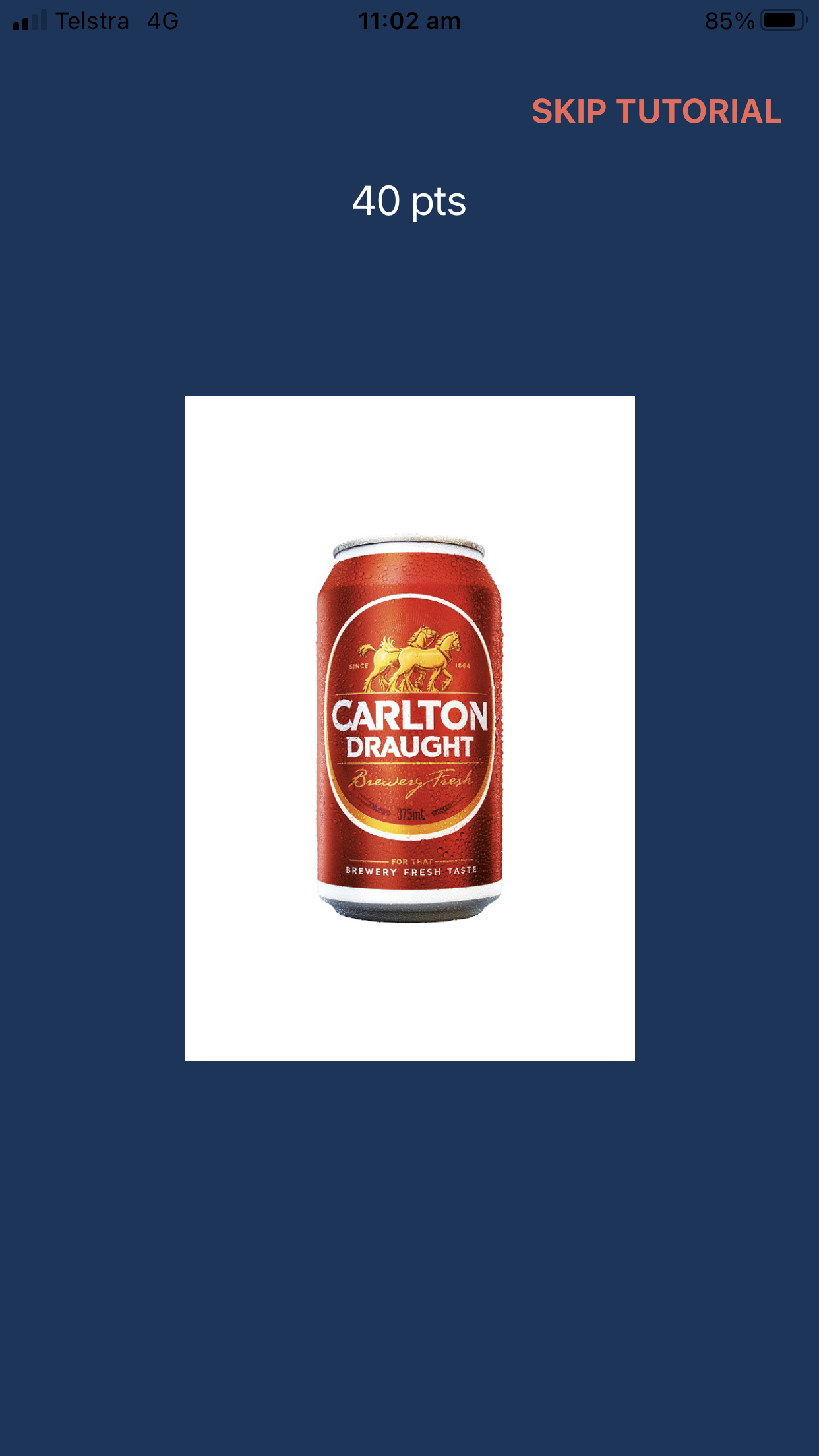

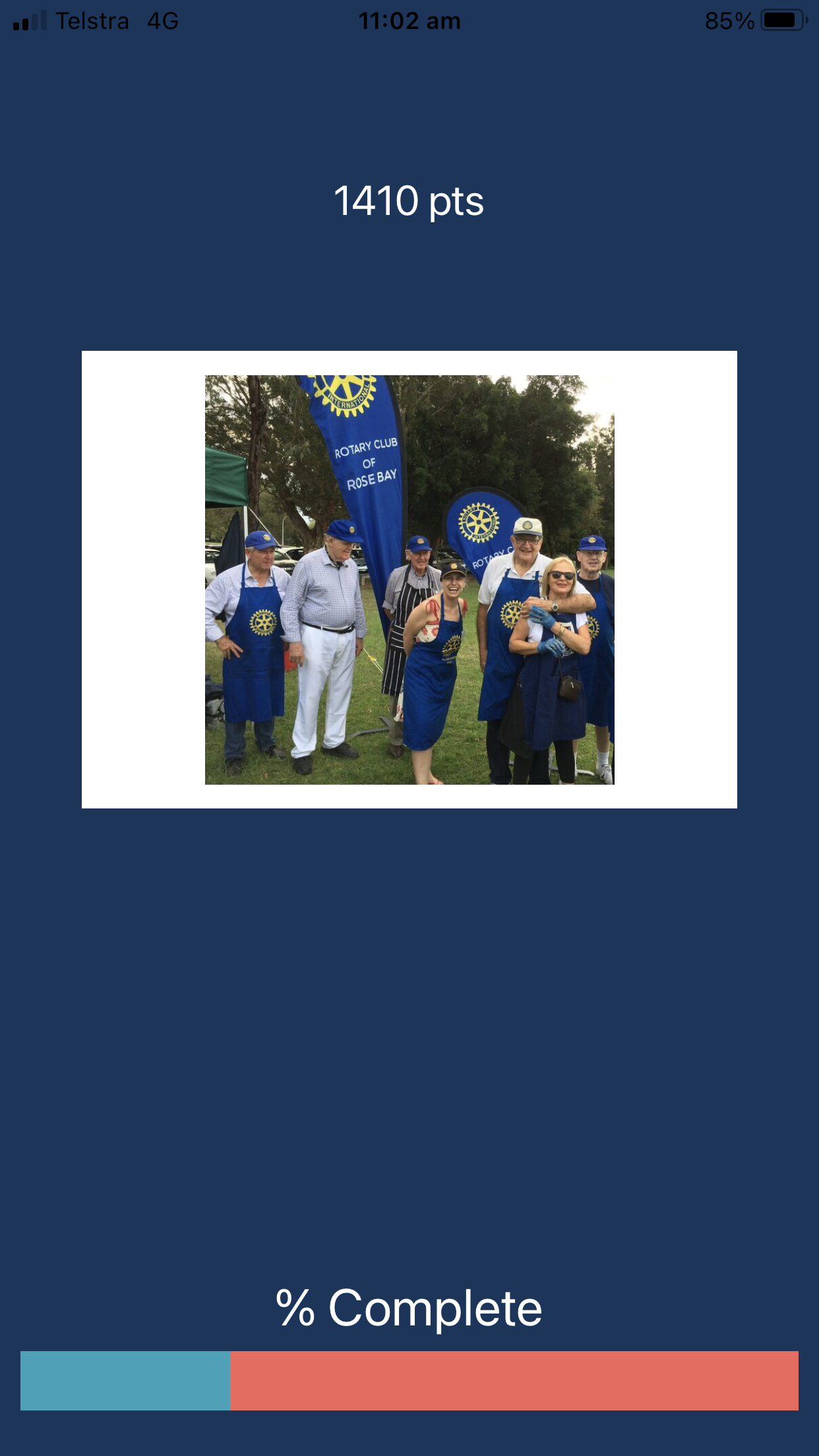


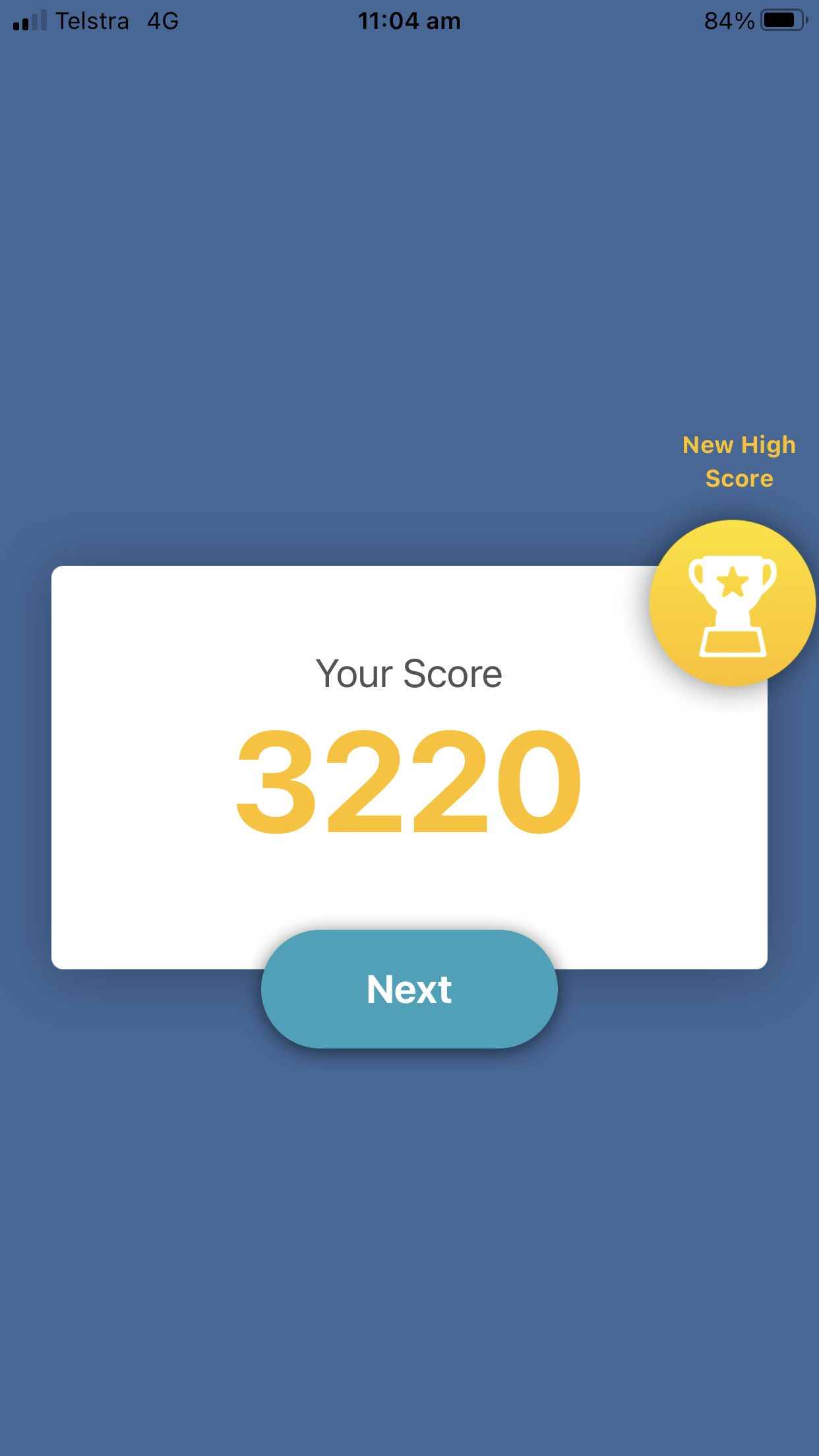

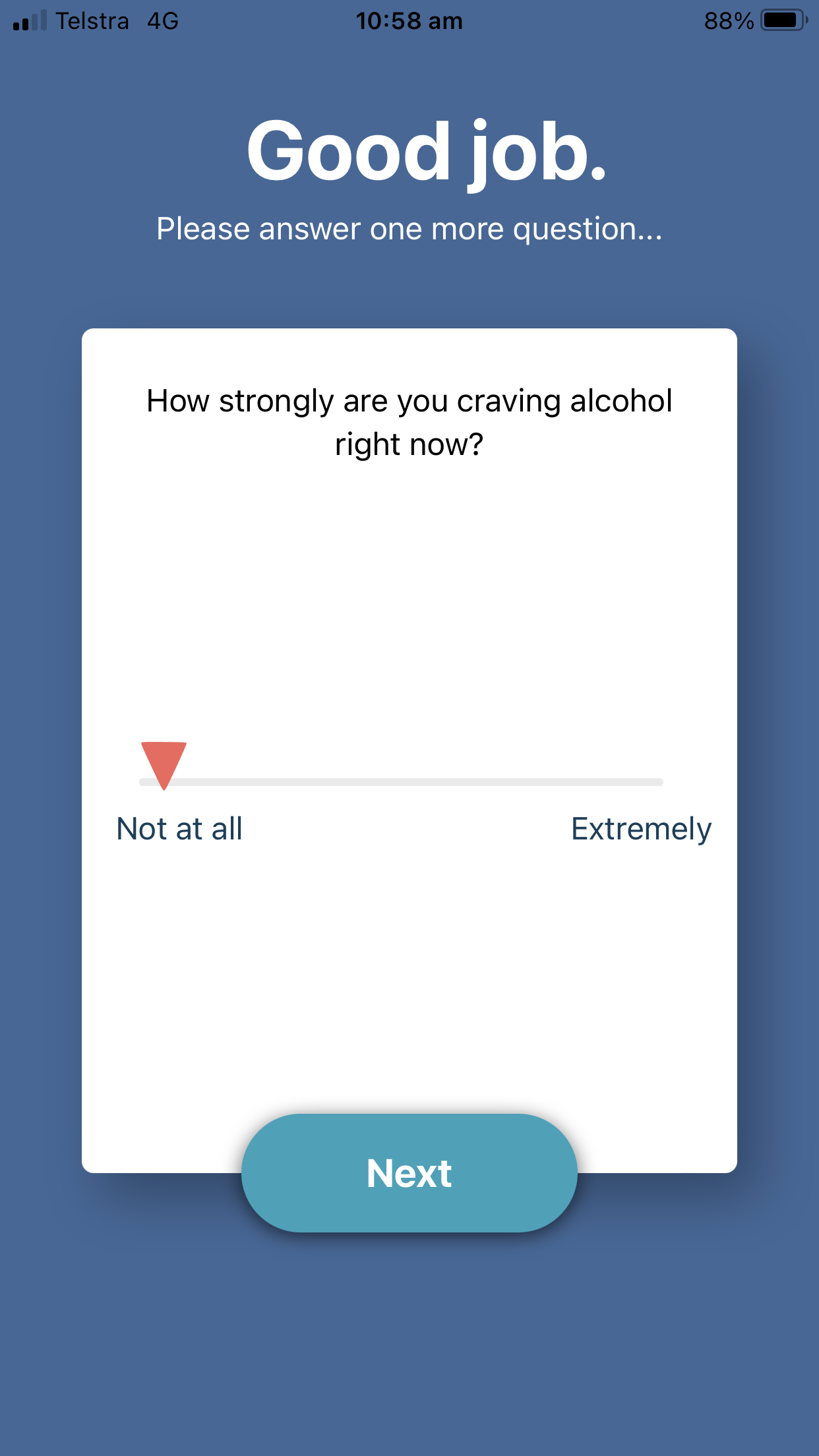


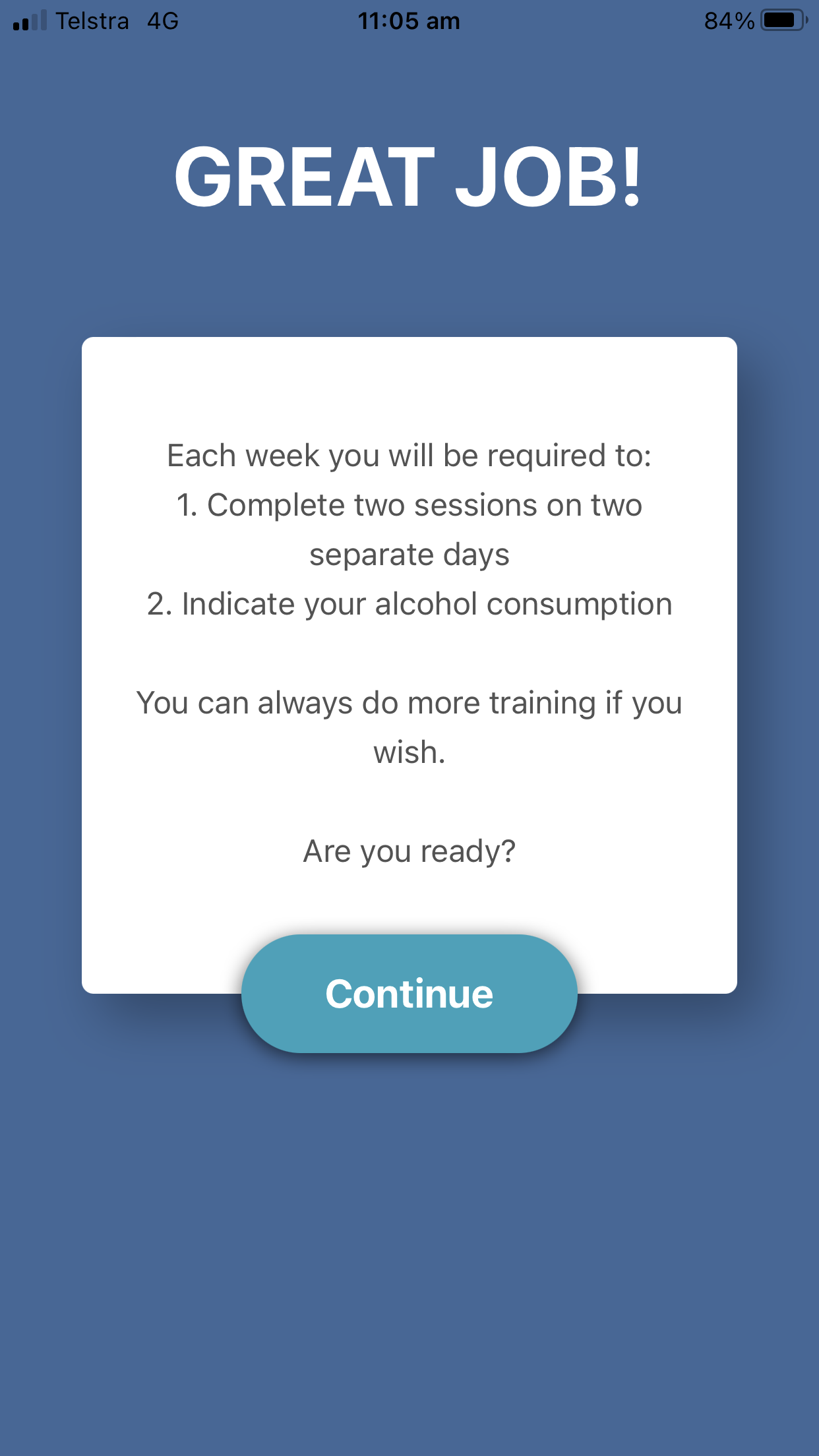

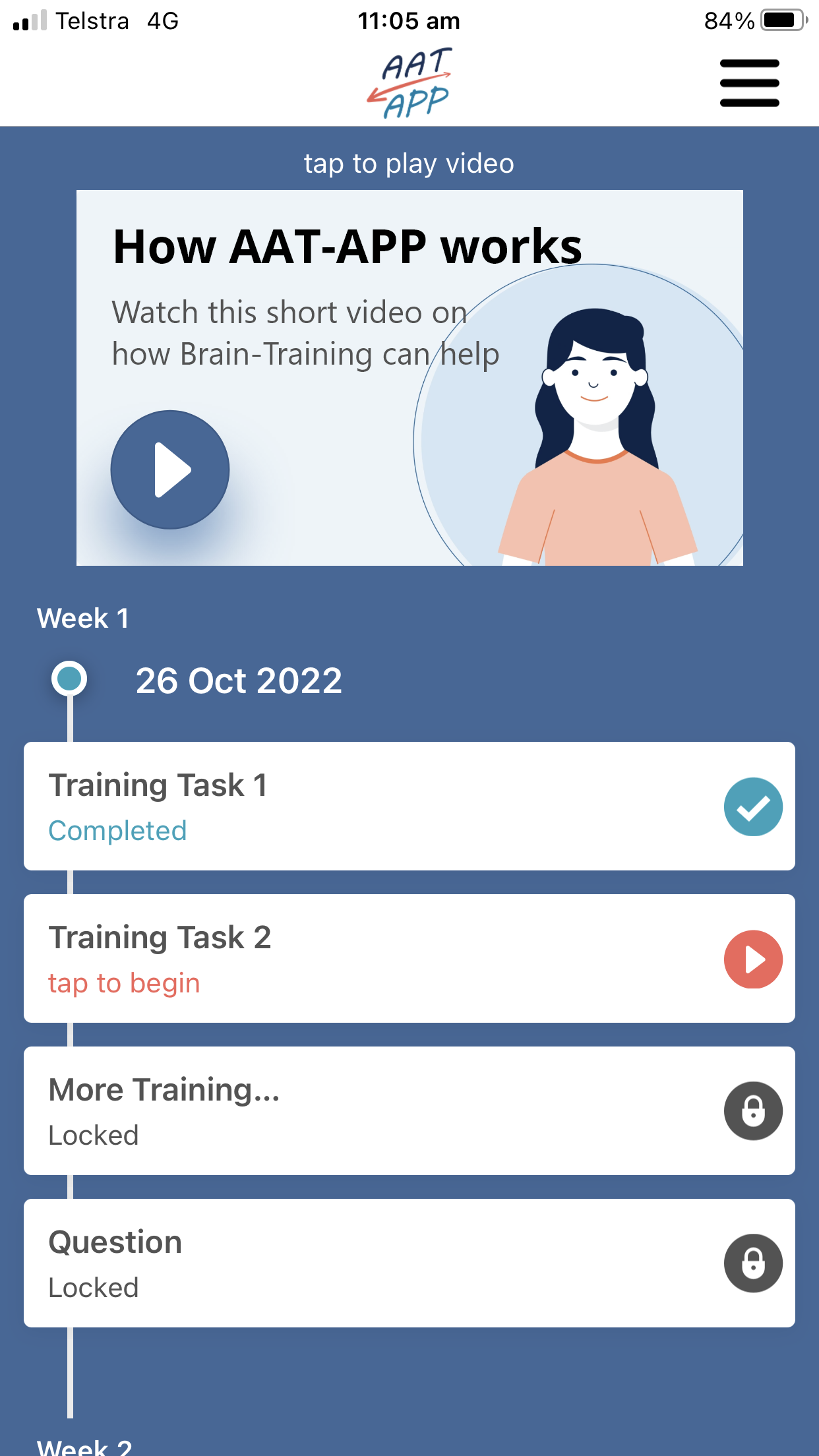

Supplement: Supplementary file 2 — Supplementary Material 2 [file 13722_2026_646_MOESM2_ESM.docx]
